# *Rev-Erbα* and Photoreceptor Outer Segments modulate the Circadian Clock in Retinal Pigment Epithelial Cells

**DOI:** 10.1038/s41598-019-48203-3

**Published:** 2019-08-13

**Authors:** Nemanja Milićević, Nadia Mazzaro, Ivanka de Bruin, Esmée Wils, Jacoline ten Brink, Anneloor ten Asbroek, Jorge Mendoza, Arthur Bergen, Marie-Paule Felder-Schmittbuhl

**Affiliations:** 1 Centre National de la Recherche Scientifique, Université de Strasbourg, Institut des Neurosciences Cellulaires et Intégratives (UPR 3212), 67000 Strasbourg, France; 2 Department of Clinical Genetics, Amsterdam UMC, University of Amsterdam, Meibergdreef 9, 1105 AZ Amsterdam, The Netherlands; 3 Department of Ophthalmology, Amsterdam UMC, University of Amsterdam, Meibergdreef 9, 1105 AZ Amsterdam, The Netherlands; 4 Netherlands Institute for Neuroscience (NIN-KNAW), Amsterdam, The Netherlands

**Keywords:** Molecular neuroscience, Circadian mechanisms, Neurophysiology

## Abstract

Retinal photoreceptor outer segments (POS) are renewed daily through phagocytosis by the adjacent retinal pigment epithelial (RPE) monolayer. Phagocytosis is mainly driven by the RPE circadian clock but the underlying molecular mechanisms remain elusive. Using ARPE-19 (human RPE cell-line) dispersed and monolayer cell cultures, we investigated the influence of cellular organization on the RPE clock and phagocytosis genes. PCR analysis revealed rhythmic expression of clock and phagocytosis genes in all ARPE-19 cultures. Monolayers had a tendency for higher amplitudes of clock gene oscillations. In all conditions *ARNTL*, *CRY1*, *PER1-2*, *REV-ERBα*, *ITGB5*, *LAMP1* and *PROS1* were rhythmically expressed with *REV-ERBα* being among the clock genes whose expression showed most robust rhythms in ARPE-19 cells. Using RPE-choroid explant preparations of the *mPer2*^*Luc*^ knock-in mice we found that *Rev-Erbα* deficiency induced significantly longer periods and earlier phases of PER2-bioluminescence oscillations. Furthermore, early phagocytosis factors β_5_-Integrin and FAK and the lysosomal marker LAMP1 protein levels are rhythmic. Finally, POS incubation affects clock and clock-controlled phagocytosis gene expression in RPE monolayers in a time-dependent manner suggesting that POS can reset the RPE clock. These results shed some light on the complex interplay between POS, the RPE clock and clock-controlled phagocytosis machinery which is modulated by *Rev-Erbα*.

## Introduction

The mammalian retina contains a complex circadian clock system which is independent from the central ‘master’ clock located in the suprachiasmatic nucleus (SCN) of the brain^1^. Retinal circadian clocks consist of layer-specific, coupled oscillators^2^. On a molecular level, the circadian clock consists of interlocking feedback loops composed of about a dozen transcription factors, with a core positive loop composed of CLOCK and BMAL1, and negative regulatory loops formed by REV-ERBα and β, Period 1–3 (PER 1–3) and Cryptochrome 1,2 (CRY1,2) proteins^3^. The retina has a unique feature as a peripheral oscillator that can adjust its daily physiology according to changes in light intensity^4–6^.

Numerous physiological, cellular and biochemical processes in the retina are regulated by circadian clocks including melatonin secretion, dopamine synthesis, sensitivity of ion channels, visual pigment synthesis, melanopsin expression and POS removal^7^. In particular, photo-oxidized POS removal and phagocytosis by the RPE, is a highly rhythmic process occurring as a daily peak in which 10% of their volume is shed^8^. The importance of POS clearance is highlighted by a number of retinal pathologies caused by deficits in RPE phagocytosis. For example, DNA mutations of *MERTK* cause RPE phagocytosis defects leading to photoreceptor degeneration^9^. Despite decades of research, it is still unclear whether photoreceptors themselves or the interactions between the photoreceptors and the RPE initiate rhythmic phagocytosis^7^. Recent evidence indicates that RPE possesses a circadian oscillator^10,11^, but its potential role in regulating the phagocytosis machinery remains unknown.

The removal of damaged POS is performed by RPE apical microvilli that project into the interphotoreceptor matrix. They surround and ultimately engulf POS^12^. These interactions are facilitated by the unique cellular organization of the RPE which is maintained by a complex intercellular cytoskeletal network^13^. Culturing conditions significantly influence cellular morphology and circadian rhythms in the SCN^14^, cardiomyocytes^15^ and hepatocytes^16^. Whether the cytoskeletal network is necessary for sustaining rhythmicity of the RPE remains an open question. The daily clearance of POS makes the RPE one of the most phagocytically active cells in the human body^17^. This imposes a high metabolic burden in the RPE, in particular at the beginning of the light phase of the light/dark (LD) cycle, during peak phagocytic activity. It is not entirely clear if the RPE copes with this proteolytic load by rhythmic lysosomal biogenesis^18^.

In this study, we investigated whether clock-controlled phagocytosis and clock gene expression profiles are dependent on the structure and organization of the RPE. Next, we investigated the impact of *Rev-Erbα* deletion on the RPE clock using PER2::LUC bioluminescence recordings. We found that the ARPE-19 monolayers show rhythms in the POS phagocytosis machinery, and that POS might in turn entrain the RPE clock.

## Results

### Dispersed ARPE-19 cells show a circadian rhythm of clock and phagocytosis gene expression

**Figure 1:**
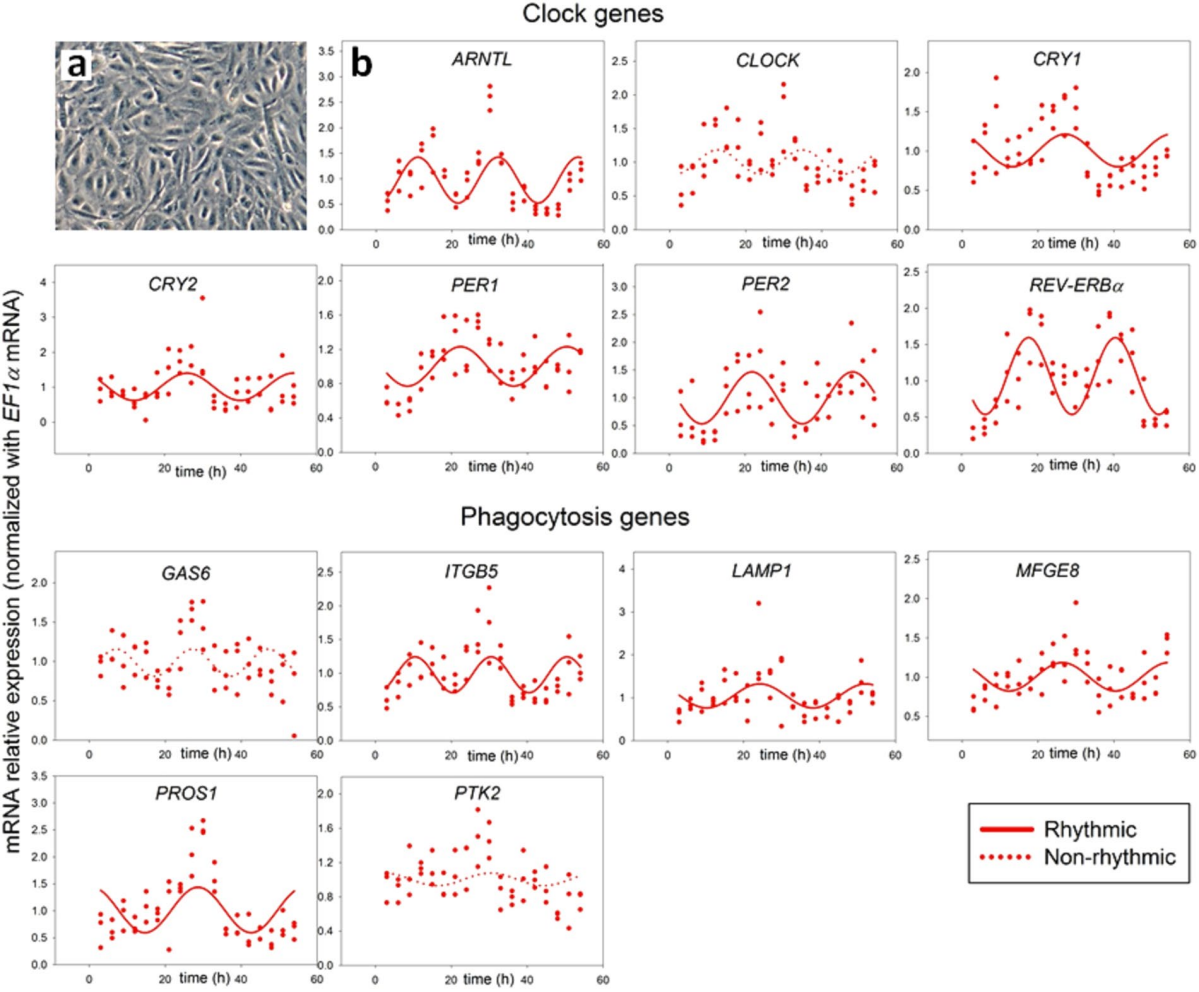
(**a**) Light microscopy image of ARPE-19 dispersed cell cultures. (**b**) In serum-shocked ARPE-19 dispersed cell cultures rhythmic expression was observed for clock (*PER1*, *PER2*, *CRY1*, *CRY2*, *REV-ERBα* and *ARNTL*) and phagocytosis (*ITGB5*, *MFGE8* and *PROS1*) genes (n = 3). Traces represent the best fitted sinusoidal regressions supporting rhythmic gene expression (dashed lines when non-rhythmic expression).

Since cultured RPE monolayers reflect physiological conditions, we hypothesized that the monolayer cellular organization would enhance the rhythmic gene expression profile of RPE cells. To test this, we first cultured ARPE-19 as dispersed cells (Fig. 1a). We harvested ARPE-19 cells at 3 h intervals for 54 h after initial serum-shock and we characterized the circadian expression profile of clock and phagocytosis genes by RT-PCR (Fig. 1b). Results of non-linear regression analysis showed that the dispersed cells exhibited rhythmic expression of the following clock genes: *ARNTL*, *CRY1*, *CRY2*, *PER1*, *PER2*, *REV-ERBα*, and the phagocytosis genes: *ITGB5*, *LAMP1*, *MFGE8* and *PROS1*. Our results suggest that ARPE-19 cells contain a robust oscillator (Table 1). Dispersed cells did not display rhythmic expression of the clock gene *CLOCK* and phagocytosis genes *GAS6* and *PTK2* (Fig. 1b; Table 1).

**Table 1:** Statistical analysis of clock and phagocytosis gene mRNA rhythmicity in ARPE-19 dispersed cells and monolayers.

Non-linear regression analysis					1-way ANOVA	
Gene	P value	Period (h)	Acrophase (h)	Amplitude	P value	F value
**Dispersed ARPE-19 cells**						
**Clock genes**						
*ARNTL*	**0**.**0003**	21.10 ± 0.98	11.01 ± 1.04	0.45 ± 0.09	<**0**.**0001**	18.11
*CLOCK*	0.1493	22.24 ± 2.06	12.61 ± 2.14	0.18 ± 0.08	**0**.**0002**	4.07
*CRY1*	**0**.**0432**	28.00 ± 2.84	27.14 ± 1.46	0.21 ± 0.07	<**0**.**0001**	4.99
*CRY2*	**0**.**0072**	28.00 ± 2.30	25.90 ± 1.20	0.39 ± 0.11	**0**.**0056**	2.73
*PER1*	**0**.**0007**	28.00 ± 1.92	22.40 ± 1.09	0.23 ± 0.05	<**0**.**0001**	5.09
*PER2*	<**0**.**0001**	26.47 ± 1.33	21.79 ± 0.87	0.47 ± 0.09	**0**.**0140**	2.39
*REV-ERBα*	<**0**.**0001**	22.86 ± 0.77	17.64 ± 0.60	0.53 ± 0.07	<**0**.**0001**	6.82
**Phagocytosis genes**						
*GAS6*	0.0699	20.00 ± 1.38	7.11 ± 1.86	0.17 ± 0.06	**0**.**0064**	2.68
*ITGB5*	**0**.**0021**	20.00 ± 0.96	10.46 ± 1.14	0.27 ± 0.06	<**0**.**0001**	4.84
*LAMP1*	**0**.**0265**	28.00 ± 2.70	24.24 ± 1.44	0.28 ± 0.09	**0**.**0108**	2.48
*MFGE8*	**0**.**0112**	28.00 ± 2.40	26.39 ± 1.24	0.18 ± 0.05	**0**.**0002**	4.05
*PROS1*	**0**.**0029**	28.00 ± 2.10	0.62 ± 2.36	0.42 ± 0.11	<**0**.**0001**	12.69
*PTK2*	0.6378	28.00 ± 6.44	2.38 ± 7.23	0.07 ± 0.06	**0**.**0007**	3.55
**ARPE-19 monolayers**						
**Clock genes**						
*ARNTL*	**0**.**0234**	20.48 ± 1.57	12.63 ± 1.64	0.36 ± 0.11	**0**.**0066**	2.60
*CLOCK*	0.1745	27.94 ± 3.49	10.69 ± 2.55	0.28 ± 0.12	0.3330	1.17
*CRY1*	**0**.**0187**	20.54 ± 1.11	6.36 ± 1.44	0.26 ± 0.08	**0**.**0023**	2.97
*CRY2*	0.1784	26.28 ± 2.64	20.93 ± 1.83	0.24 ± 0.11	**0**.**0485**	1.89
*PER1*	**0**.**0025**	26.59 ± 1.78	18.29 ± 1.29	0.53 ± 0.13	**0**.**0349**	2.01
*PER2*	**0**.**0005**	21.86 ± 1.07	19.39 ± 0.84	0.85 ± 0.19	0.0611	1.84
*REV-ERBα*	**0**.**0024**	20.84 ± 1.11	14.70 ± 1.12	0.40 ± 0.10	**0**.**0165**	2.27
**Phagocytosis genes**						
*GAS6*	0.5007	28.00 ± 5.11	24.45 ± 2.79	0.12 ± 0.08	0.5375	0.94
*ITGB5*	**0**.**0295**	28.00 ± 2.75	23.07 ± 1.52	0.27 ± 0.09	**0**.**0038**	2.87
*LAMP1*	**0**.**0479**	23.61 ± 1.70	11.29 ± 1.73	0.43 ± 0.15	0.1712	1.44
*MFGE8*	0.1796	28.00 ± 3.42	21.93 ± 2.01	0.21 ± 0.09	0.5430	0.94
*PROS1*	**0**.**0030**	28.00 ± 2.04	19.38 ± 1.35	0.44 ± 0.11	**0**.**0033**	2.84
*PTK2*	**0**.**0219**	28.00 ± 2.40	21.23 ± 1.45	0.25 ± 0.08	**0**.**0044**	2.74

Data were fitted to the equation y = y_0_ + c**⋅**cos[2π**⋅**(t − φ)/τ] by non-linear regression. Bold values represent p < 0.05.

### ARPE-19 monolayers show a circadian rhythm of clock and phagocytosis gene expression

**Figure 2:**
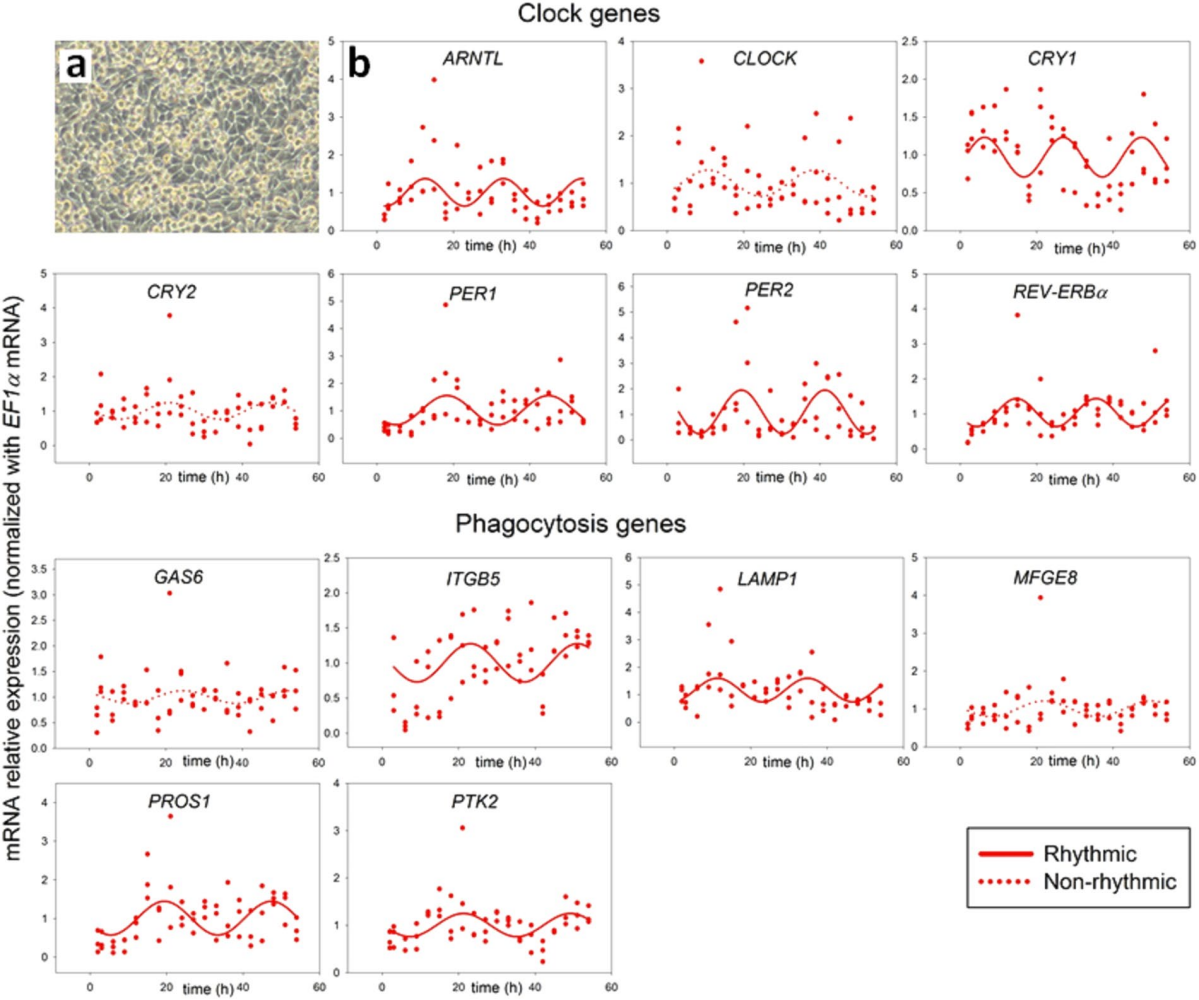
(**a**) Light microscopy image of ARPE-19 monolayers. (**b**) In serum-shocked ARPE-19 monolayers rhythmic expression was observed for clock (*PER1*, *PER2*, *CRY1*, *REV-ERBα* and *ARNTL*) and phagocytosis (*ITGB5*, *LAMP1*, *PTK2* and *PROS1*) genes (n = 3). Traces represent the best fitted sinusoidal regressions supporting rhythmic gene expression (dashed lines when non-rhythmic expression).

To reveal if the physiology of the monolayer cellular organization influences the rhythmic expression profile of ARPE-19 cells, we next performed an RT-PCR analysis of ARPE-19 monolayers (Fig. 2a) harvested at 3 h intervals following serum-shock, also during 54 h (Fig. 2b). Monolayers showed rhythmic expression of clock genes: *ARNTL*, *CRY1*, *PER1*, *PER2*, *REV-ERBα* and phagocytosis genes: *ITGB5*, *LAMP1*, *PROS1* and *PTK2*. We did not observe rhythmic expression of clock genes: *CLOCK* and *CRY2* or the phagocytosis genes: *MFGE8* and *GAS6*. The distinct feature of the ARPE-19 monolayer gene expression profile as compared to dispersed cells is the rhythmic expression of *PTK2*. Further, results of non-linear regression fitting indicate that amplitudes of clock gene oscillation in serum-shocked monolayers are on average higher than in serum-shocked dispersed cells (Table 1). These results corroborate our initial hypothesis. Thus, further experiments in this study were performed using RPE monolayers.

### Is the rhythmicity of the phagocytosis machinery of ARPE-19 monolayers translated to the protein level?

**Figure 3:**
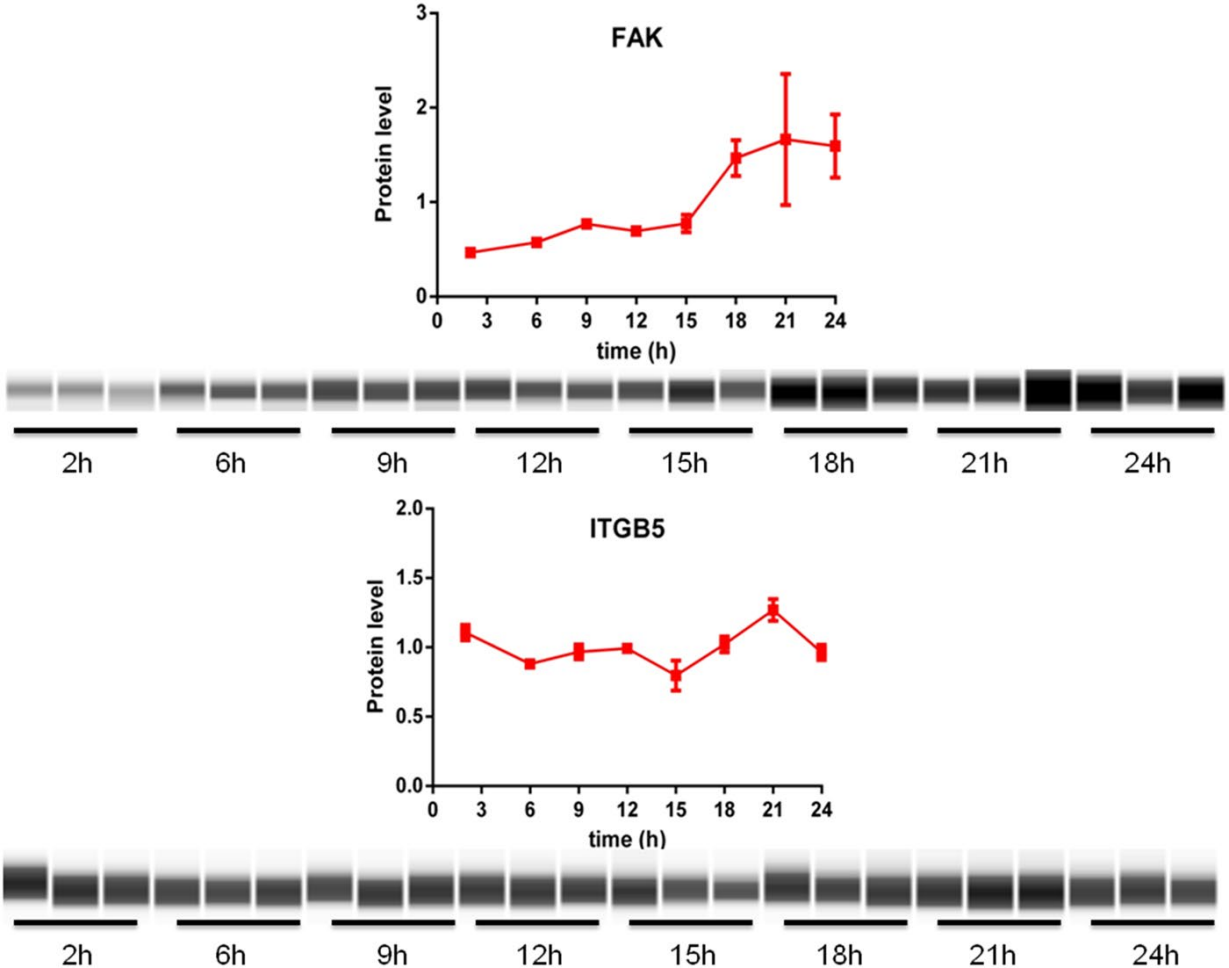
Expression profile of FAK and ITGB5 proteins in serum-shocked ARPE-19 monolayers over 24 h. FAK (F(7,16) = 2.98, p < 0.05) and ITGB5 levels (F(7,16) = 5.06, p < 0.01) were affected by time. Uncropped digitally generated WES™ images are provided in Supplementaty material (Figs S6–11). Values are shown as means ± SEM (n = 3).

To investigate whether ARPE-19 monolayers also show oscillations in phagocytosis protein levels we used the novel capillary based quantitative Wes™ system^19^. We first assessed total protein concentration of each sample using a modified Lowry reaction (DC protein assay, see methods) and loaded 0.6 µg of protein extracts from each sample for Wes™ analysis. Using the Wes™ apparatus we found that the levels of the commonly utilized reference protein in circadian experiments, β-ACTIN, tended to differ over 24 h (Fig. S3). Conversely, non-normalized WES results showed very little variability in the levels of proteins of interest within each time-point (Fig. 3), which substantially increased after β-ACTIN normalization (not shown). Thus, we analyzed raw protein data without β-ACTIN correction (Fig. 3). Time had an effect on the product of *PTK2*, FAK (1-way ANOVA, F(7,16) = 2.98; p < 0.05) and on ITGB5 (1-way ANOVA, F(7,16) = 5.06; p < 0.01) protein levels in serum-shocked monolayers. Non-linear regression revealed that FAK (p < 0.01) protein levels were rhythmic in synchronized monolayers, whereas ITGB5 protein levels may show a tendency to do so (p = 0.11). As controls, we evaluated the expression of these proteins in monolayers which were not synchronized and found that levels of FAK (1-way ANOVA, F(7,16) = 2.26; p = 0.084) and ITGB5 (1-way ANOVA, F(7,16) = 0.84; p = 0.57) proteins did not vary over 24 h (Fig. S3). In addition, in non-synchronized monolayers non-linear regression showed that FAK (p = 0.05) protein levels were at the limit of significance for rhythmicity, but not ITGB5 protein levels (p = 0.35). These results suggest that, in agreement with rhythmic mRNA levels, proteins of the POS phagocytosis machinery show rhythmic expression.

### *Rev-Erbα* modulates the period and the phase of RPE molecular oscillations, but is dispensable for sustaining the clockwork of the RPE

**Figure 4:**
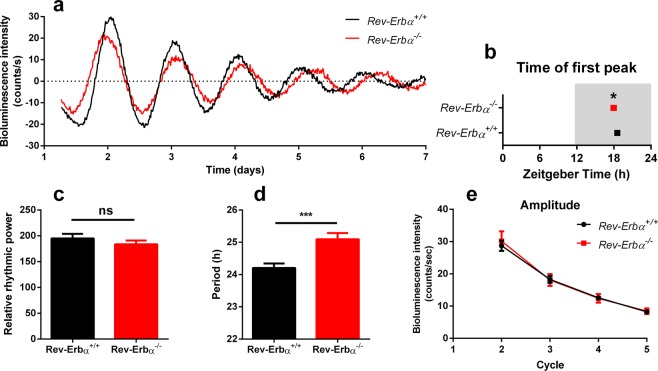
A *Rev-Erbα* knock-out *in vivo* lengthens the period and advances the phase in RPE molecular rhythms and gives modest overall impairment of RPE rhythms. (**a**) Representative PER2::LUC bioluminescence traces recorded from *Rev-Erbα*^+/+^ *and Rev-Erbα*^-/-^ RPE-choroid explants. The first obvious peak of PER2::LUC bioluminescence (around 48 h after start of the experiment) in *Rev-Erbα*^-/-^ RPE-choroid occurred earlier than in wild type samples (**b**, *t*-test of log_10_ transformed data, p < 0.05). Mutant samples also displayed longer periods (**d**, *t*-test, p < 0.001). Relative rhythmic power (**c**, *t*-test, ns) and ampli*t*ude of successive peaks (**e**, 2-way ANOVA, genotype F(1,26) = 0.0283, ns) did not differ between genotypes. Values are shown as means ± SEM (n = 14–15). Time in b is projected ZT with ZT12 = lights off, *p < 0.05, ***p < 0.001.

Based on p-values of non-linear regression and 1-way ANOVA analysis, we conclude from our *in vitro* data that among the clock genes *REV-ERBα* was exhibiting the most significant rhythmicity throughout culture conditions (Table 1). We therefore hypothesized that it is a major element of the RPE clockwork and might be indispensable for maintaining RPE molecular rhythms. To assess this hypothesis, we studied the RPE clock in *Rev-Erbα* knock-out animals maintained on the *mPer2*^*Luc*^ background. PER2::LUC bioluminescence recordings revealed that *Rev-Erbα* deficient RPE-choroids had longer periods (25.10 ± 0.19 h) (Fig. 4d) than controls (24.20 ± 0.14 h) and had an earlier phase (Fig. 4b). Conversely, *Rev-Erbα* deficiency did not affect relative rhythmic power (Fig. 4c) and amplitude (Fig. 4e). Thus, these results suggest that *Rev-Erbα* is involved in the RPE clock, most likely as a fine-tuning element.

### A POS challenge has a phase dependent effect on clock and clock-controlled phagocytosis in the RPE

**Figure 5:**
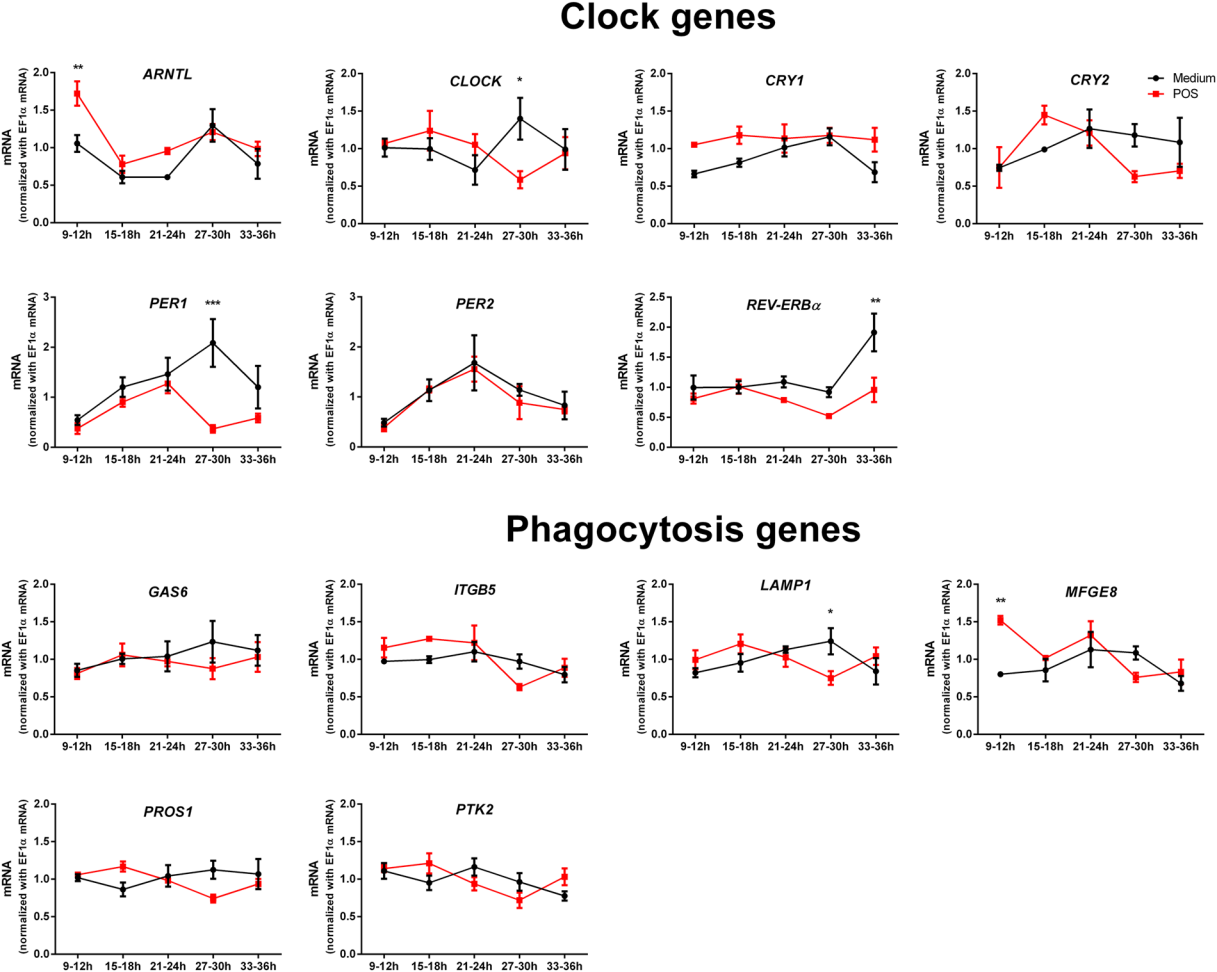
A 3 h POS incubation affects clock and phagocytosis gene expression in ARPE-19 monolayers at specific time-phase intervals. Cells were synchronized by adding 50% bovine serum for 2 h (t = 0–2 h) and medium was changed at t = 2 h. Over the following 1.5 days cultures were challenged by 3 h exposure to POS (red lines) either between t = 9–12 h, between t = 15–18 h, between t = 21–24 h, between t = 27–30 h or between t = 33–36 h. Treatment with medium at identical time interval was used as a control (black lines). Cells were harvested immediately after the 3 h incubation. At specific time-points, POS significantly affected the expression profile of clock genes: *ARNTL*, *CLOCK*, *PER1* and *REV-ERBα* and phagocytosis genes: *LAMP1* and *MFGE8*. Values are shown as means ± SEM (n = 3). Analysis was performed by two-way ANOVAs followed by Holm-Sidak’s post-hoc test. *p < 0.05, **p < 0.01, ***p < 0.001.

Removal of photo-oxidized POS is a crucial function of the RPE that in turn might influence the RPE gene expression profile^20,21^. Previous transcriptomics studies examined the effect of POS incubation on the RPE and ARPE-19 mRNA expression profile^20,21^. However, to the best of our knowledge, transcriptomics databases showed no major effects of POS on mRNA expression levels of clock (except *Per2*)^21^ and phagocytosis genes^20,21^. We tested if POS incubation can affect the RPE-clock and phagocytosis genes by applying POS obtained from bovine eyes for 3 h at defined time-points on synchronized ARPE-19 monolayers (Fig. 5). Cells were immediately harvested after a POS/medium challenge. Two-way ANOVA analysis revealed that treatment significantly affected the expression of *ARNTL* (F(1,20) = 9.95, p < 0.01), *CRY2* (F(1,20) = 13.14, p < 0.01), *PER1* (F(1,20) = 14.14, p < 0.01), *REV-ERBα* (F(1,20) = 12.73, p < 0.01) and *MFGE8* (F(1,20) = 4.88, p < 0.05). Specifically, post-hoc testing of POS vs. medium effects showed that POS when applied at t = 9–12 h increased the expression of *ARNTL* (p < 0.01) and *MFGE8* (p < 0.01). POS administration at t = 27–30 h decreased the expression of *CLOCK* (p < 0.05), *PER1* (p < 0.001) and *LAMP1* (p < 0.05), whereas administration at t = 33–36 h increased *REV-ERBα* expression (p < 0.01). Overall, these data reveal that POS affect gene expression in the ARPE-19 monolayers in a time-dependent manner. Such effect was not observed in non-synchronized ARPE-19 monolayers, in which a 3 h and 6 h POS challenge did not induce any change in clock and phagocytosis gene expression (Fig. S4). Thus, exposure to POS might reset the RPE-clock.

### Does the circadian clock regulate the lysosomal processing of RPE phagosomes?

LAMP1 is a constituent of lysosomal membranes and is present in most lysosomes^22^. Considering that our RT-PCR results showed that *LAMP1* mRNA is rhythmic (Figs 1 and 2, Table 1) and affected by POS in a time-dependent manner (Fig. 5), we hypothesized that the circadian clock regulates lysosomal POS processing which is in turn affected by POS.

**Figure 6:**
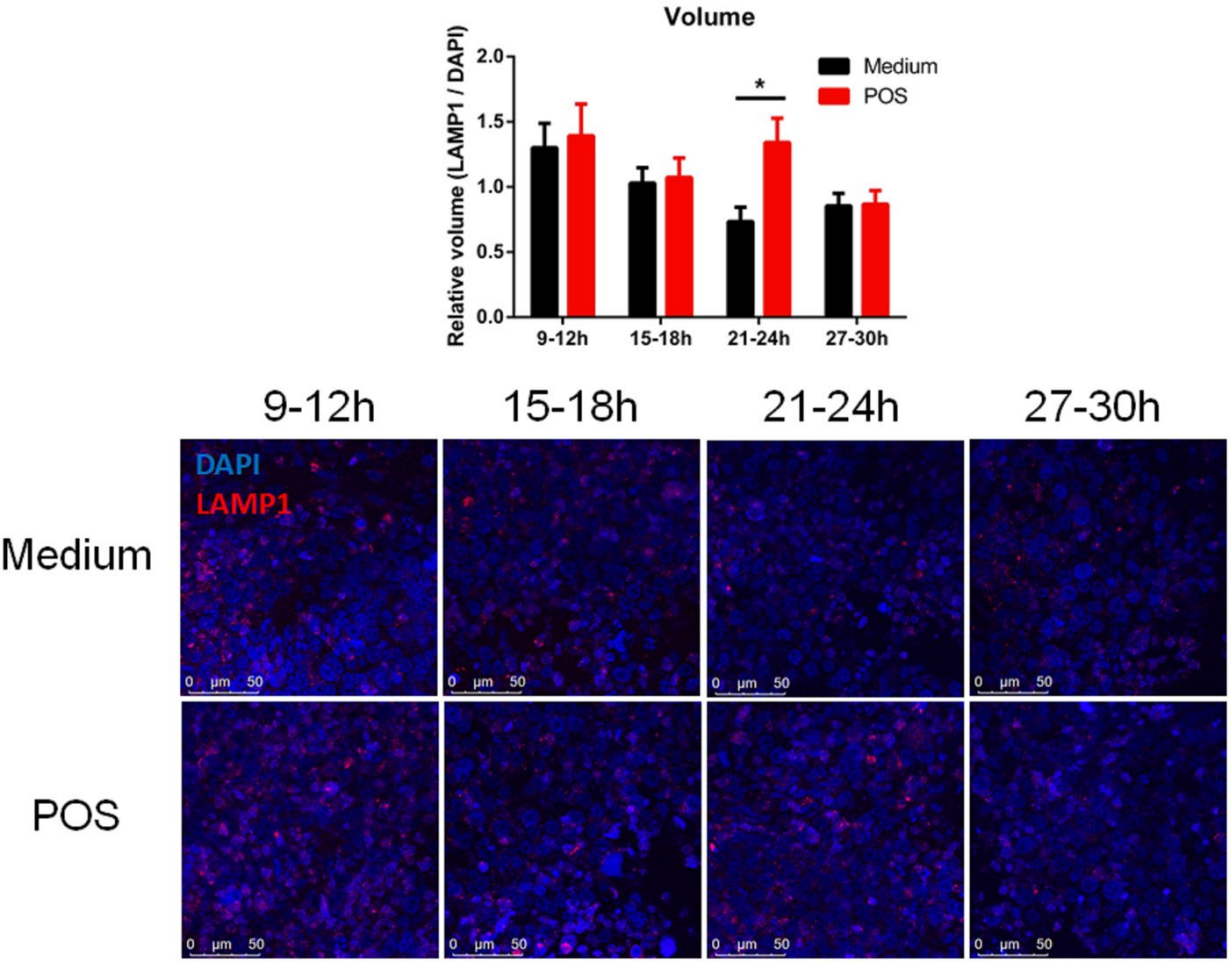
Circadian regulation of lysosomal POS processing. ARPE-19 monolayers were serum-shocked (t = 0–2 h), challenged with bovine POS or medium for 3 h at defined time-points and immediately fixed. Lysosomes were visualized with the lysosomal marker LAMP1. (Top) Quantification of z-stacks revealed that time affects the total stained volume of LAMP1/DAPI (2-way ANOVA, F(3,287) = 3.30; p < 0.05). Holm-Sidak’s post-hoc test revealed that POS induced a higher volume of LAMP1/DAPI at t = 21–24 h (p < 0.05). (Bottom) Representative confocal images show that a POS incubation at t = 21–24 h induces a stronger LAMP1 signal vs. medium challenged controls. Values are shown as means ± SEM (n = 10 culture inserts/treatment/time-point; total number of z-stacks = 32–42/treatment/time-point).

To test this possibility, we incubated synchronized ARPE-19 monolayers with POS at different times of the circadian phase. The effect of 3 h POS incubation on the expression of the lysosomal marker LAMP1 was assessed at 6 h intervals by immunohistochemistry and confocal analysis. We used Leica Application Suite software (see Methods) for quantifying LAMP1 relative to the nuclear marker DAPI signal: the relative volume (Fig. 6), fluorescence intensity sums (Fig. S5) and particle counts (Fig. S5). Time had a significant effect on LAMP1 levels for all outcome parameters in medium challenged controls (Figs 6 and S5; 1-way ANOVA: relative volume F(3, 137) = 3.31; p < 0.05; relative counts F(3, 130) = 3.39; p < 0.05; relative intensity sums F(3, 136) = 13.44; p < 0.0001). In addition, by using the cosinor-derived non-linear regression analysis, we observed that the levels of LAMP1 were rhythmic in medium challenged cells (relative volume p < 0.05; relative counts p < 0.05; relative intensity sums p < 0.0001), suggesting that lysosomal levels are regulated by the circadian clock.

Finally, we observed that a POS challenge performed at t = 21–24 h increased LAMP1 levels (Figs 6 and S5; Holm-Sidak’s post-hoc test: relative volume p < 0.05; relative counts p < 0.05) relative to medium controls. Although POS treatment did not significantly increase LAMP1/DAPI relative intensity sums (p = 0.57), the trend was the same as for relative volume and counts (Fig. S5). Taken together, our results corroborate the hypothesis that the POS processing machinery is under circadian control and responds to POS in a phase-dependent manner.

## Discussion

The phagocytosis of POS is a temporally controlled event occurring as a unique peak during the LD cycle, roughly an hour after light onset^23,24^. Recent findings suggest the importance of phagocytosis peak timing for retinal health. Namely, mice deficient in melatonin receptors (MT1^-/-^ and MT2^-/-^)^25^, the Ca^2+^-dependent K^+^ (BK^-/-^) channel^26^ and the integrin receptor ITGB5^27^ had altered timing of POS phagocytosis and displayed symptoms of retinal degeneration. Despite extensive efforts, the circadian clock driving the peak in POS phagocytosis is not characterized. One possibility is that the circadian oscillator located in retinal photoreceptors^2,28^ initiates downstream signalling cascades that expose phosphatidylserine “eat-me” signals on POS. Another possibility is that the RPE circadian oscillator interacts with photoreceptors via a hitherto unidentified pathway. In this paper, we studied the regulation of the RPE oscillator and the influence of retinal POS on the RPE. We describe the rhythmic expression profile of clock and phagocytosis genes in ARPE-19 cell cultures. Our results demonstrate that *Rev-Erbα* modulates the RPE clock. We found that the interaction with POS has a time-dependent effect on the expression of clock and phagocytosis genes in the RPE. In summary, our data suggest that the control of periodic phagocytosis of POS involves complex regulations which involve both photoreceptors and RPE.

The existence of a circadian clock in the RPE was recently discovered^10^. Subsequent work has focused on the mechanisms that regulate the RPE clock^11,29–31^ and RPE-clock-directed downstream physiology^30,32^. Here, we study the rhythmic machinery of the RPE in further detail. For investigating this machinery we used the ARPE-19 cell culture model which was shown to ingest bovine/porcine POS via the MFGE8 – ITGB5 – FAK pathway^33^. This model has some limitations such as morphological and protein expression variability of ARPE-19 cells^33^, which we minimized by using similar cell batches, low passages, consistent culturing conditions and careful monitoring of transepithelial electrical resistance and microscopy. In all ARPE-19 cell cultures, we found rhythmic expression for most clock and phagocytosis genes tested except for *CLOCK* and *GAS6*. We report that *ARNTL*, *CRY1*, *PER1-2*, *REV-ERBα*, *ITGB5*, *LAMP1* and *PROS1* were rhythmically expressed in ARPE-19 regardless of culturing conditions. This suggests that the rhythmicity of their expression likely plays an important role in RPE physiology. Indeed, knock-outs of *Arntl*/*Bmal1* in mice exhibited a retinal phenotype with impaired ERG signals^34,35^ and disruption of BMAL1 function impaired phagocytosis in human RPE cell lines^30^. Similarly, *Itgb5* knock-outs are incapable of peak RPE phagocytosis activity^27^. Whereas nothing is known about *Pros1* expression in the RPE during the day/night cycle, control of this gene by the circadian clock has been described in the liver^36^ and aorta^37^. Considering that Protein S (encoded by *PROS1*) binds to phosphatidylserine “eat-me” signals on the POS membrane^38^, it might be that rhythmic secretion of Protein S by the RPE coincides with phosphatidylserine exposure on POS, thus facilitating POS uptake. Our results are also in accordance with the RNA sequencing study of Mure and coworkers (2018), that showed rhythmic expression of numerous clock and phagocytosis genes such as: *ARNTL*, *CRY1-2*, *PER1-2*, *REV-ERBα*, *LAMP1* and *ITGB5* in baboon RPE^37^. Taking our data together with those from the literature, we confirm the existence of autonomous clock machinery within RPE cells, and point to *ITGB5*, *LAMP1* and *PROS1* as RPE clock-controlled genes. The rhythmic expression of these genes likely plays an important role in the control of daily phagocytosis.

We found a tendency for enhanced circadian amplitudes and shorter periods in RPE monolayer cultures compared to dispersed cell cultures. This raises the possibility that the RPE, organized as a tight junction epithelium, enhances the rhythmicity of this tissue. The negative relationship between period length and cellular coupling strength is supported by theoretical simulations of the SCN^39^ and experimentally in the retina^2^. In agreement with our results, bioluminescence recordings of the whole retina had higher oscillation amplitudes than individual retinal layers and dissociated cells^2^. Also, monolayers uniquely show rhythmic *PTK2* expression. The protein product of *PTK2*, FAK, is an essential link between POS binding and engulfment mechanisms of integrin-mediated phagocytosis^40^. Therefore, we suggest that intercellular coupling within the monolayer morphology likely contributes to RPE clockwork and phagocytic capability by enhancing clock gene oscillation amplitudes and mobilizing rhythmic expression of the phagocytosis molecular machinery.

Recent work showed that entrainment of RPE clocks is mediated via the secreted neurotransmitter originating in the inner retina, e.g. dopamine^11^. Similarly, histaminergic and cholinergic signalling shift the phase of the RPE clock but the sources of retinal histamine and acetylcholine are not fully known^30,31^. In the retina, layer-specific circadian clocks are tightly coupled and thus maintain synchrony in the tissue^2^. Considering that the RPE and photoreceptors lay in close contact it is likely that their interaction mutually sets their clocks and thereby enables the coordination of molecular events in the phagocytic cascade. We found that POS incubation affected the expression of clock genes *ARNTL*, *CLOCK*, *PER1* and *REV-ERBα* and phagocytosis genes *MFGE8* and *LAMP1* in a time-dependent manner. Unexpectedly, this effect was absent at time-points 24 h prior/after the POS effect. The average period length of rhythmic clock gene mRNA expression in monolayers is 22.06 h (Table 1). Consequently, cultures separated by a 24 h time-gap are likely not in the same phase, and thus might not respond in the same manner. In addition, PCR analysis gives a snapshot of the transcriptional machinery at a given time. Thus, the discrepancies in results observed between 24 h time-points might also be due to limitations of temporal resolution of our PCR approach. The phase dependent effect of POS incubation on RPE mRNA expression suggests that photoreceptors may reset the RPE clocks by shedding POS that most likely bind to the ITGB5 receptors expressed on the RPE apical membrane^41^. The binding of POS to the ITGB5 receptors activates the PI3K-AKT signalling pathway^33,42^. Based on literature, we postulate that the activation of PI3K regulates the recruitment of *ARNTL*/*CLOCK* and downstream clock-regulated transcription^43^. The PI3K-AKT pathway might also recruit *MFG8*, which in our results was not rhythmic (Table 1) but was upregulated by POS treatment at the same time as *ARNTL*. Therefore, our data supports the argument that the RPE phagocytic machinery is driven by the RPE clock and reset by entrainment signals originating from the retina, potentially including POS themselves.

Recent work highlights *Rev-Erbα* as an important regulator in the retina implicated in numerous functions: the phototransduction cascade^44^, retinal development^44^, retinal light sensitivity and information processing^44,45^. In addition, *Rev-Erbα* can rescue *Nr2e3*-mediated retinal degeneration in mice^46^. In our study, one of the most rhythmically expressed clock genes in ARPE-19 cells was *REV-ERBα* (Table 1). This finding suggested that *Rev-Erbα* is a clock component necessary for sustaining RPE rhythmic oscillations. However, bioluminescence recordings revealed that *Rev-Erbα*^-/-^ RPE-choroids were capable of generating rhythmic PER2::LUC oscillations. They had earlier peaks after start of culture and longer periods, but did not differ in amplitude and rhythmic power. Thus, our results confirm that *Rev-Erbα* contributes to the precise timing of the RPE clock. In line with these observations, *Rev-Erbα* deficient mice show an overall modest clock phenotype featuring altered phase-shifting property and period length^47,48^. However, *Rev-Erbs* are functionally redundant and a deficiency of both *Rev-Erbα* and *Rev-Erbβ* results in a dramatically impaired clock phenotype and metabolic disorder^48,49^. Considering that the RPE shows rhythmic *Rev-Erbβ* expression^37^, it is likely that *Rev-Erbα*^-/-^ *–*induced impairments are compensated for by *Rev-Erbβ*.

The lysosomal associated membrane protein 1 (LAMP1) is known to be involved in the lysosomal digestion of internalized POS by the RPE and is considered as a late stage marker of POS phagocytosis^17,50^. Deficient *LAMP1* mRNA levels were found in AMD patients^51^, thus suggesting that *LAMP1* expression levels are necessary for POS processing and proper retinal function. Quantification of our confocal microscopy results indicates rhythmic levels of relative LAMP1 protein expression, volume of LAMP1 in cells and number of LAMP1 positive particles. Considering that 50% of all lysosomes express LAMPs^22^, we speculate that rhythmic mRNA expression of *LAMP1* is translated to a protein level which may lead to rhythmic lysosomal biogenesis. In earlier work, *LAMP1* mRNA was found to be rhythmic in baboon RPE^37^ and LAMP1 protein levels in mouse RPE^21^. However, in rat RPE-choroids LAMP1 protein levels did not differ over time^18^. This inconsistency may be due to close sampling intervals in which samples with peak and trough LAMP1 expression were not included^18^. Further, we found that POS incubation performed at a specific time-interval increased LAMP1 relative volume and number of LAMP1 positive particles in ARPE-19 monolayers, but not LAMP1 relative protein expression levels. These results suggest that POS can increase the biosynthesis of phagolysosomes in the RPE in a phase-dependent manner. The POS-induced increase in LAMP1 occurred slightly after the peak mRNA expression of most phagocytosis genes. Therefore, we propose that the POS internalization and processing mechanisms are tightly coordinated and regulated by the circadian clock which is in turn modulated by POS.

## Materials and Methods

### Cell culture

ARPE-19 cells were obtained from ATCC (CRL-2302, Manassas, VA, USA). Frozen cells were cultured using Dulbecco’s Modified Eagle’s Medium: Nutrient Mixture F-12 with L-glutamine and Hepes (DMEM/F12, Gibco Life Technologies, Carlsbad, CA, USA) supplemented with 10% heat-inactivated fetal calf serum (FCS) (Sigma-Aldrich, St. Louis, MO, USA) and 1% penicillin/streptomycin (PS) (Gibco BRL, Grand Island, NY, USA). Cells were cultured at 37 °C and 5% CO_2_. Medium was changed thrice a week. In all experiments we used cells of the similar passage and batch (a maximum of 6 passages after ATCC supplied ARPE-19) and monitored cell growth and morphology by microscopy. Cells were either grown as monolayers or non-monolayers (dispersed cell culture) using medium with 1% FCS (other component concentrations were not changed). Dispersed cells were plated on 12-well culture plates (#3513 Costar, Kennebunk, ME, USA) at a density of 1.6 × 10^5^ cells/cm^2^ in a volume of 1.5 ml and cultured for 4 days. Monolayers were grown by plating on transwell filters (#3460 Costar) according to Dunn *et al*.^52^, with the following modifications: filters were coated with 160 µl of a 1:40 dilution of growth-factor reduced Matrigel (Corning, NY, USA), an artificial extracellular matrix material shown to influence cell differentiation^53,54^, in DMEM F12 serum-free medium and air-dried overnight. Before seeding, the basolateral chamber of each well was filled with 1 ml of DMEM F12 medium supplemented with 1% FCS and PS. Cells were plated at a density of 1.6 × 10^5^ cells/cm^2^ in a total volume of 0.5 ml/filter.

Monolayers were cultured for 28 days and morphology was confirmed by immunostaining with the tight-junction marker ZO-1 (Fig. S1). Transepithelial electrical resistance (TEER) was measured to ensure that ARPE-19 cells established intact monolayers prior to inclusion in experiments. TEER measurements were performed using an ohmmeter (Millicell ERS-2, Billerica, MA, USA) and corrected for background by subtracting the value of a Matrigel coated filter without cells and multiplied by transwell filter area. A TEER of 20 Ωcm^2^ was arbitrarily regarded as the threshold value for an intact monolayer^55^. To exclude any possible confounding effects of Matrigel in PCR and Western blotting experiments, Matrigel was dissolved from the cells by incubating in Cell Recovery solution (Corning, Bedford, MA, USA) for 30 min at 4 °C. In addition, we performed PCR reactions (data not shown) and Western blots (Fig. S2) on inserts coated with Matrigel without cells.

Synchronization was performed with the ‘serum-shock’ procedure using 50% FCS in DMEM/F12 between time-points 0–2h^56^. The non-synchronized cells used as controls received no medium change for 3 days prior to the start of the experiment and throughout the sampling period.

### RNA isolation, cDNA synthesis and mRNA quantification

Total RNA was extracted using the RNeasy mini kit (Qiagen, Valencia, CA, USA) according to the manufacturer’s instructions. Complementary DNA was synthesized from 50 ng of total RNA using oligo (d_._T) primed reactions with Superscript III reverse transcriptase (Life technologies, Waltham, MA, USA). The synthesized cDNA was amplified with transcript-specific, intron-spanning primers with PCR amplification cycles optimized for product quantification. Primer sequences are provided in the Supplementary Table S1. PCR products were electrophorezed on 2% agarose gels containing ethidium-bromide and images were captured using the FujiFilm LAS300 (Tokyo, Japan). Bands were quantified using Aida image analyzer v4.26. Band intensities for each gene were divided with the expression value of the non-rhythmic reference gene *Ef1α*. We used average gene expression values within an experiment as a calibrator to normalize between experiments.

### Western blotting

After Matrigel removal, cells were lysed with RIPA buffer (10 mM Tris/HCl pH 8, 140 mM NaCl, 1 mM EDTA, 0.5 mM EGTA, 0.1% SDS, 0.1% Na-deoxycholate, 1% Triton X-100) supplemented with protease (c0mplete, Roche, Mannheim, Germany) and phosphatase inhibitor mixture (PhosSTOP, Roche). Protein concentration was determined using the modified Lowry assay (DC protein assay, Bio-Rad laboratories, USA). All samples were diluted to 0.2 mg/ml and 0.6 μg of sample protein extract was loaded into each Wes™ well plate. Western blots were performed using a Wes™ Simple Western capillary-based automated immunoblot system (ProteinSimple, San Jose, CA, USA) according to the manufacturer’s instructions (Supplementary Table S2).

### Immunofluorescence and confocal microscopy

Cells were fixed with 4% paraformaldehyde for 10 min, blocked and permeabilized using PBS with 10% normal donkey serum, 1% bovine serum albumin (BSA), 0.2% Triton X-100 for 1 h. Cells were incubated with primary antibodies (Supplementary Table S2) for 90 min, then with Cy3 conjugated secondary antibodies (Jackson ImmunoResearch, USA) for 1 h. All antibodies were diluted in PBS supplemented with 1% BSA and 0.2% Triton X-100. Samples were mounted with Vectashield antifade medium with DAPI (Vector laboratories, Burlingame, CA, USA). Confocal stacks were acquired with a Leica TSC SP-8 mounted on a Leica DMI6000 inverted microscope using a HC Plan Apochromat x63 objective. We quantified LAMP1 and DAPI signals using Leica Application Suite software (LAS X 3D ver. 3.3.0, Leica Microsystems CMS GmbH, Wetzlar, Germany) with the goal of quantifying LAMP1 protein levels and visualizing phagolysosomes. To minimize bias, we reported all meaningful outcome parameters of quantification: fluorescence intensity, volume of staining and stained particle counts. Fluorescence intensity is defined as the sum of the gray-scale values of all voxels belonging to LAMP1 and DAPI stained objects. The sum of LAMP1/DAPI fluorescence intensities represents the relative LAMP1 protein expression level. Volume of staining is the volume of all voxels covered by the LAMP1 and DAPI stained objects in the image in µm³. This parameter represents the relative volume LAMP1 occupies in cells. Particle counts are the total number of objects in the image and represent the number of LAMP1 positive particles in cells (likely phagolysosomes^22^). All parameters are presented as ratios of LAMP1/DAPI. For each culture insert 5 or more z-stacks were captured. We screened automatically and for quantification purposes, we excluded z-stacks that had low cell numbers. In our analysis we used z-stacks that contained at least 25% DAPI volume fill. From 3 independent experiments we gathered a total of 401 z-stacks and excluded 104 (74% of total were used for quantification). We used average values of volume, sum of fluorescence intensity and counts within an experiment as a calibrator to normalize between experiments.

### Photoreceptor outer segment isolation

Bovine eyes were obtained from the slaughterhouse. Eyes were cut open and retinas were scrapped from posterior eye cups. Retinas were homogenized in 42% sucrose, 1 M NaCl, 0.1 M MgCl_2_ and 1 M Tris-acetate. The supernatant was centrifuged, filtered through a sterile gauze and diluted in 2 volumes of 0.01 M Tris-acetate pH 7.4. POS were isolated in a sucrose gradient by ultracentrifugation (Optima ultracentrifuge, Beckman Coulter, Inc., Brea, CA) at 25,000 RPM for 40 min at 4 °C. POS were recovered in 0.01 M Tris-acetate, ultracentrifuged at 20,000 RPM for 30 min at 4 °C, and dissolved in DMEM:F12 medium. POS particles were counted using a haemocytometer and diluted to 1.2 × 10^8^ particles/ml. In all phagocytosis assays cells were incubated with 1.2 × 10^7^ POS particles for 3 h at 37 °C in 5% CO_2_. Although the addition of phagocytosis ligands or 10% FCS in the POS medium is advised^18,21,33,57–61^, we have not added these components because our cell culture demonstrated the ability to secrete ligands necessary for phagocytosis (Fig. S2). Furthermore, the addition of serum is known to affect the circadian clock and thus might have a confounding effect on our experiments^56,62^.

### Animals, tissue preparation and bioluminescence

Mice were bred and housed in a 12 h/12 h Light/Dark (LD) cycle (light intensity approx. 300 lux). Animals had access to water and food *ad libitum*, and were handled according to the French law implementing the European Union Directive 2010/63/EU. Experimental protocol was approved by the Animal Use and Care Committee from Strasbourg (CREMEAS; AL/08/15/02/13). We used male and female mice from our *Rev-Erbα*^-/-^ colony maintained on a homozygous *mPer2*^*Luc*^ C57BL6/J background^63^. *Rev-Erbα*^+/+^ *and Rev-Erbα*^-/-^ littermates were generated by breeding of heterozygotes. Mice were euthanized around ZT8, the eyes were removed, placed in iced HBSS and then dissected. After the cornea, lens and vitreous humour were removed, the retina and RPE-choroid cup were separated. The posterior eye cup containing the RPE-choroid was flattened using four small corner cuts, and then placed on a semi-permeable membrane (PICMORG50 Millipore Billerica, MA, USA) in a 35 mm Petri dish with 1 ml of 199 medium (Sigma-Aldrich) containing 10 mM HEPES (pH 7.2), 0.1 mM D-Luciferin potassium salt (Promega, Fitchburg, WI, USA), 25 U/mL antibiotics (PS, Sigma-Aldrich) and B27 (2%; Gibco BRL). RPE-choroid explants were placed —RPE cell layer up—on the culture membrane. Dishes were well sealed with Dow Corning high vacuum grease (Midland, MI, USA) and kept at 37 °C inside a Lumicycle (Actimetrics, Wilmette, IL, USA). Samples were recorded during at least 7 days: photons were counted during 1 min 48 sec every 15 min. Raw bioluminescence data were detrended by using a 24 h running average. Period and phase of bioluminescence oscillations were determined by using LM sin-fit (damped) function of the Lumicycle Analysis software (Actimetrics). Rhythmic power was determined by using the periodogram function of the software.

### Statistics

Data were obtained from at least 3 biological replicates and expressed as mean ± SEM. Plots were generated using GraphPad Prism software (La Jolla, CA, USA) or SigmaPlot (Systat Software, San Jose, CA, USA). Normality of distribution was confirmed using the Kolmogorov-Smirnov test. Circadian gene expression profiles were determined by nonlinear least squares fitting to a sine wave function using SigmaPlot: y = y0 + c ∙ cos [2π (t-ϕ)/τ], where τ represents period, ϕ acrophase and c amplitude. The function featured the following constraints: 20 h < τ < 28 h; ϕ < τ, ϕ > 0 and c > 0. Further analyses, where indicated, were performed using 1-way or 2-way ANOVA analysis followed by Holm-Sidak’s post-hoc tests.

## Supplementary information

Rev-Erbα and Photoreceptor Outer Segments modulate the Circadian Clock in Retinal Pigment Epithelial Cells

## Data Availability

Data supporting the conclusions of this article are included within the article and are available from the corresponding author on reasonable request.
