# Supplementary material for: Rev-Erbα and Photoreceptor Outer Segments modulate the Circadian Clock in Retinal Pigment Epithelial Cells
Source: Sci Rep. 2019 Aug 13;9:11790. doi: 10.1038/s41598-019-48203-3 (PMC6692399; doi:10.1038/s41598-019-48203-3)
Supplement: Supplementary file 1 — Rev-Erbα and Photoreceptor Outer Segments modulate the Circadian Clock in Retinal Pigment Epithelial Cells [file 41598_2019_48203_MOESM1_ESM.pdf]

Rev-Erbα and Photoreceptor Outer Segments modulate the Circadian Clock in Retinal Pigment Epithelial Cells

Nemanja Milićević[1,2], Nadia Mazzaro[1], Ivanka de Bruin[2], Esmée Wils[2], Jacoline ten Brink[2], Anneloor ten Asbroek[2], Jorge Mendoza[1], Arthur Bergen[2,3,4‡], Marie-Paule Felder-Schmittbuhl[1‡*]

[1] Centre National de la Recherche Scientifique, Université de Strasbourg, Institut des Neurosciences Cellulaires et Intégratives (UPR 3212), 67000 Strasbourg, France

[2] Departments of Clinical Genetics and [3] Ophthalmology, Amsterdam UMC, University of Amsterdam, Meibergdreef 9, 1105 AZ, Amsterdam, the Netherlands

[4] Netherlands Institute for Neuroscience (NIN-KNAW), Amsterdam, the Netherlands

‡ Equal contribution

***** Corresponding author: feldermp@inci-cnrs.unistra.fr

Supplementary material

Supplementary methods

Labelling of isolated photoreceptor outer segments

Centrifuged POS (8000 RPM for 4 min at 4°C) were labeled with 1 mg/ml FITC (Molecular Probes, Invitrogen, Carlsbad, CA) in 10 mM Na_2HPO_4, 0.1 M NaCl and rotated for 1h at RT. Excess FITC was removed by centrifugation (8000 RPM for 4 min at 4°C) and resuspension in culture medium (DMEM:F12). Labelled POS were counted by a haemocytometer and diluted to 1,2 x 10^8 particles/ml. In all phagocytosis assays cells were incubated with 1,2 x 10^7 POS particles for 3h or 6h at 37°C in 5% CO_2.

Gene	Forward Primer	Reverse Primer	Product length (bp)
	Clock genes		
ARNTL	GGAGGGACTCCAGACATTCC	GAGGAAACACTGGAGCAGGC	459
CLOCK	CTGCTCCTGTAGCTTGTGGG	ACTGCTGGAACTTTCCCTCC	494
CRY1	GGTCTTCTGGCATCAGTACC	CCCACCACTGAGACCAGTGC	275
CRY2	CCCAAACGCAAGCTGGAAGC	CGGGAAACAGCACTGGC	352
PER1	CATCCATTCGGGTTACGAAG	TTGTGGATAGCCAGCATGAG	201
PER2	GCGTCATGATGACGTACCAG	TCGCTGAGTCCCAGAGAAGG	265
REV-ERBα	TAAGCCGCACCACCTACAGC	GGCCAGAGGCTCATCTTGGA	494
	Phagocytosis genes		
GAS6	CGGAATCTGGTCATCAAGG	GAGCCACGACTTCTACTTCC	370
ITGB5	CCCAACGCCATGACCATCCT	TTCTCTGTGGTGCCTACCTAGG	360
LAMP1	CCTACAAGGAATCCAGTTG	CCTTGAAAGCCTGGACCCACA	191
MFGE8	GGCAGCAGTAAGATCTTCCCTGG	GTGCTGCCTCTGAACACCCT	273
PROS1	TGCACGTCAGTCAACTAATGCTT	TTGCATGGCAGAGGACTACACT	84
PTK2	GACATTATTGGCCACTGTGG	GCGTGAGAGCAGCAGTCAGCAT	680
	Reference gene		
EF1α	AAATAAGCGCCGGCTATGCC	CAAAGCGACCCAAAGGTGGAT	219

Supplementary table S1. List of primers used

Protein	Host species	Catalog number	Company	Technique	Dilution
β-ACTIN	Mouse	AB6276	Abcam	WES	1:50
FAK	Rabbit	06-543	Millipore	WES	1:10
ITGB5	Mouse	SC130379	Santa cruz	WES	1:10
LAMP1	Mouse	328602	Biolegend	IHC	1:150
PROS1	Rabbit	AF4036	R&D systems	WES	0,2 µg/ml
ZO-1	Rabbit	61-7300	Zymed	IHC	1:100

Supplementary table S2. List of antibodies used

Supplementary results

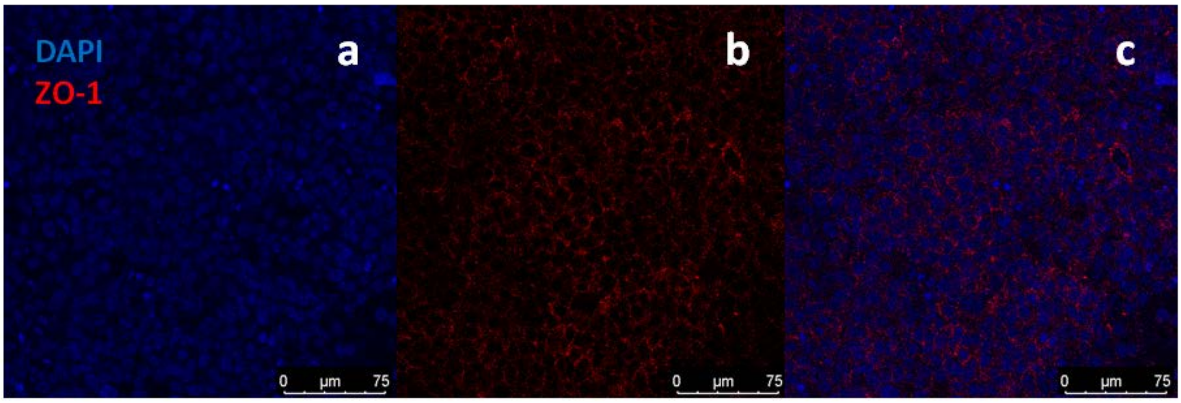

Supplementary figure S1. Polarized ARPE-19 monolayers display characteristic cobblestone morphology. (A, C) Nuclei were stained with DAPI and (B, C) tight junctions were stained with anti-ZO-1 antibody.

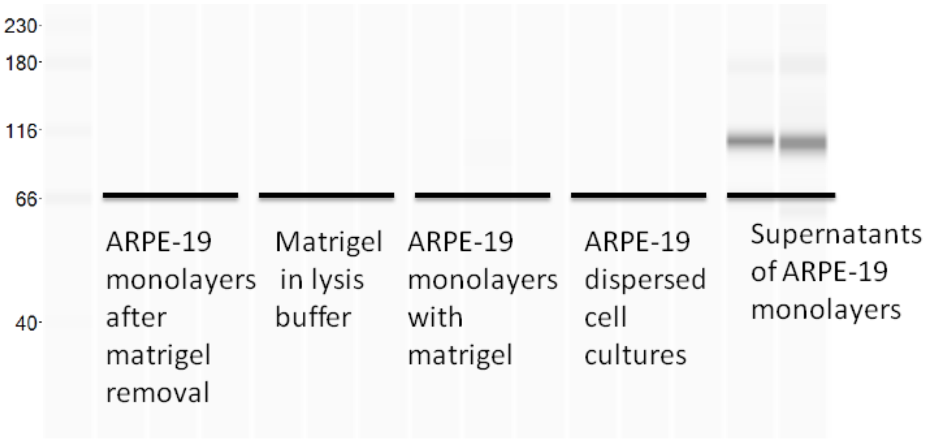

Supplementary figure S2. Protein S is secreted by ARPE-19 monolayers. Protein S was detected in serum-free supernatants of ARPE-19 monolayers, but not in ARPE-19 dispersed cell and monolayer lysates. Protein S was not detected in matrigel in lysis buffer. The uncropped WES^{TM} digitally generated image is shown in Fig. S12.

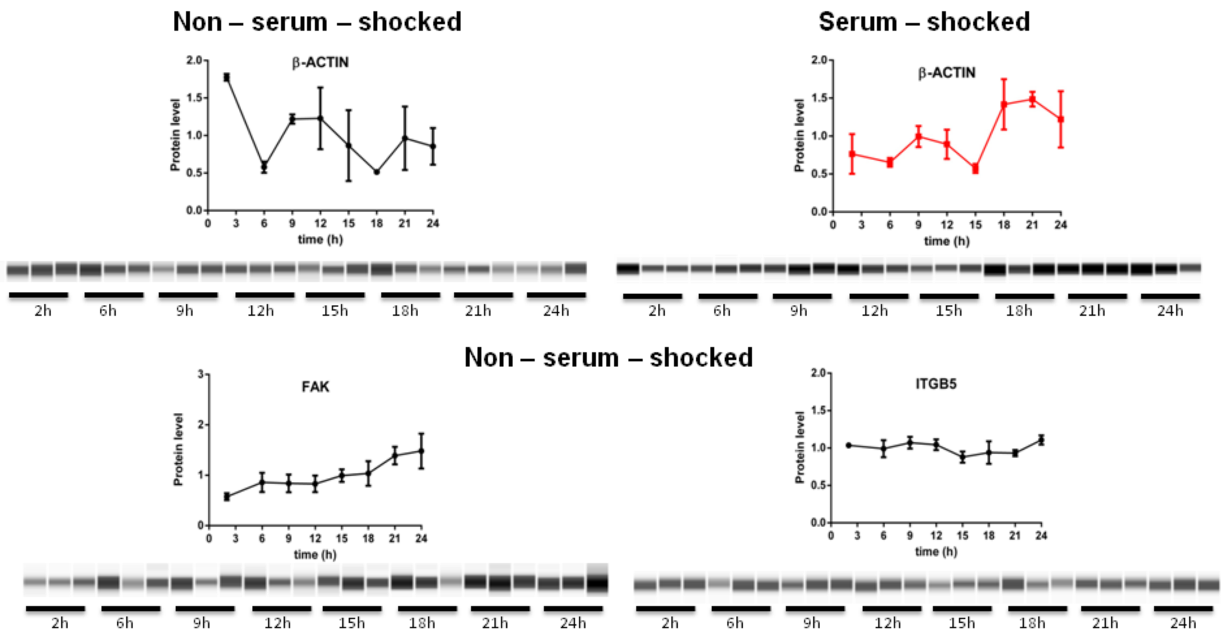

Supplementary figure S3. Protein expression profile of ARPE-19 monolayers over 24h. (Top) ARPE-19 monolayers showed unstable β-ACTIN protein levels across time-points. One-way ANOVA analysis showed that duration tended to affect β-ACTIN protein levels in monolayers that received no medium change (black trace, $F(7,16)=2.063$, $p=0.10$) and serum-shocked monolayers (red trace, $F(7,16)=2.438$, $p=0.07$). Therefore, we did not normalize using β-ACTIN in further WES experiments. (Bottom) In monolayers that were not synchronized levels of FAK (1-way ANOVA, $F(7,16) = 2.26$; $p= 0.084$) and ITGB5 (1-way ANOVA, $F(7,16) = 0.84$; $p = 0.57$) proteins did not vary over 24h. Values are shown as means±SEM (n=3). Uncropped digitally generated WES^{TM} images are provided in Fig. S6-8, 10.

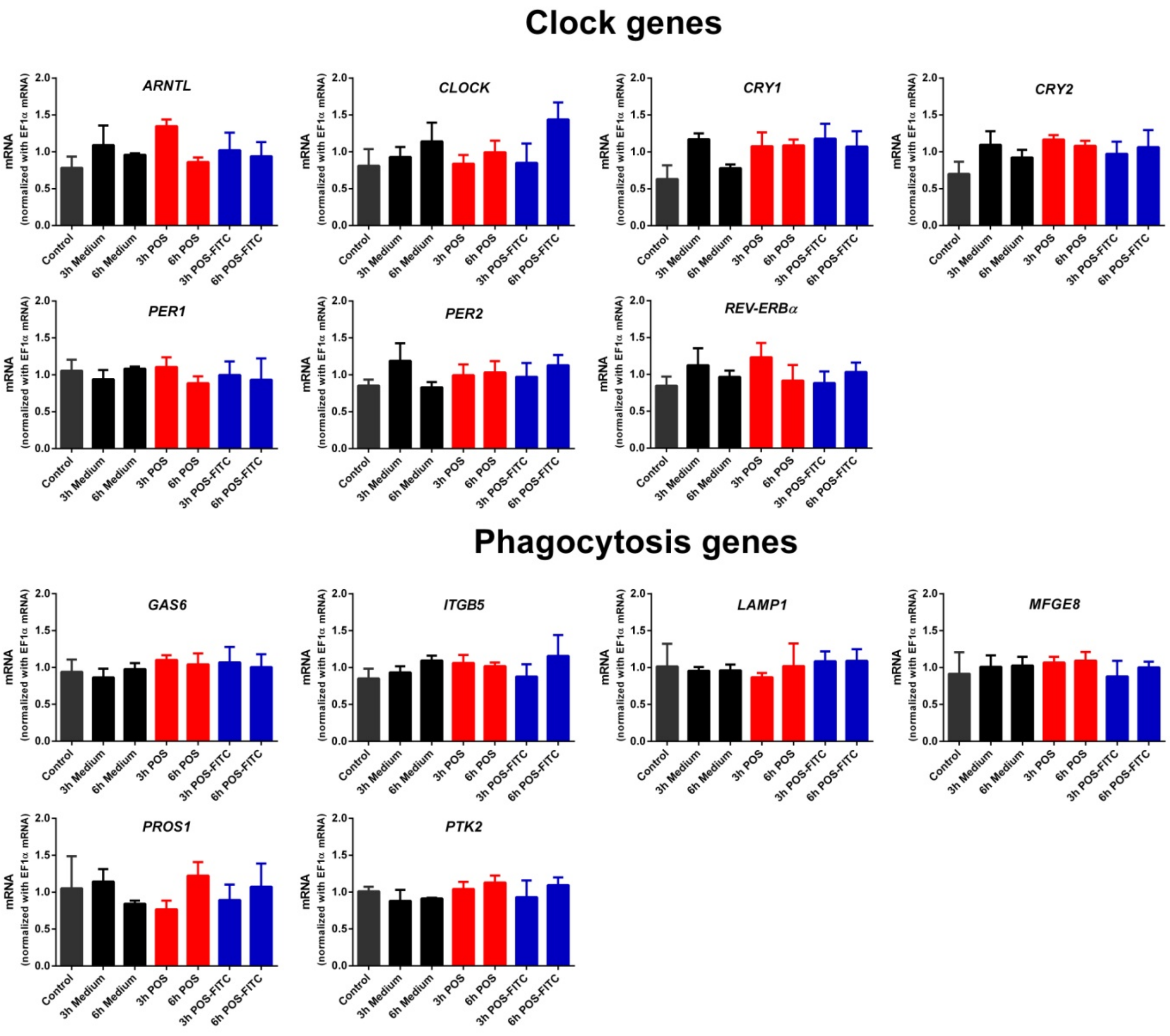

Supplementary figure S4. POS incubation does not significantly affect clock and phagocytosis gene expression in non-shocked ARPE-19 monolayers. ARPE-19 monolayers were incubated with medium, POS or labelled POS (POS-FITC) for 3 or 6h. Two-way ANOVA tests revealed no effect of treatment or duration. The experiment was repeated 3 times. The representative values are shown as means ± SEM (n=3).

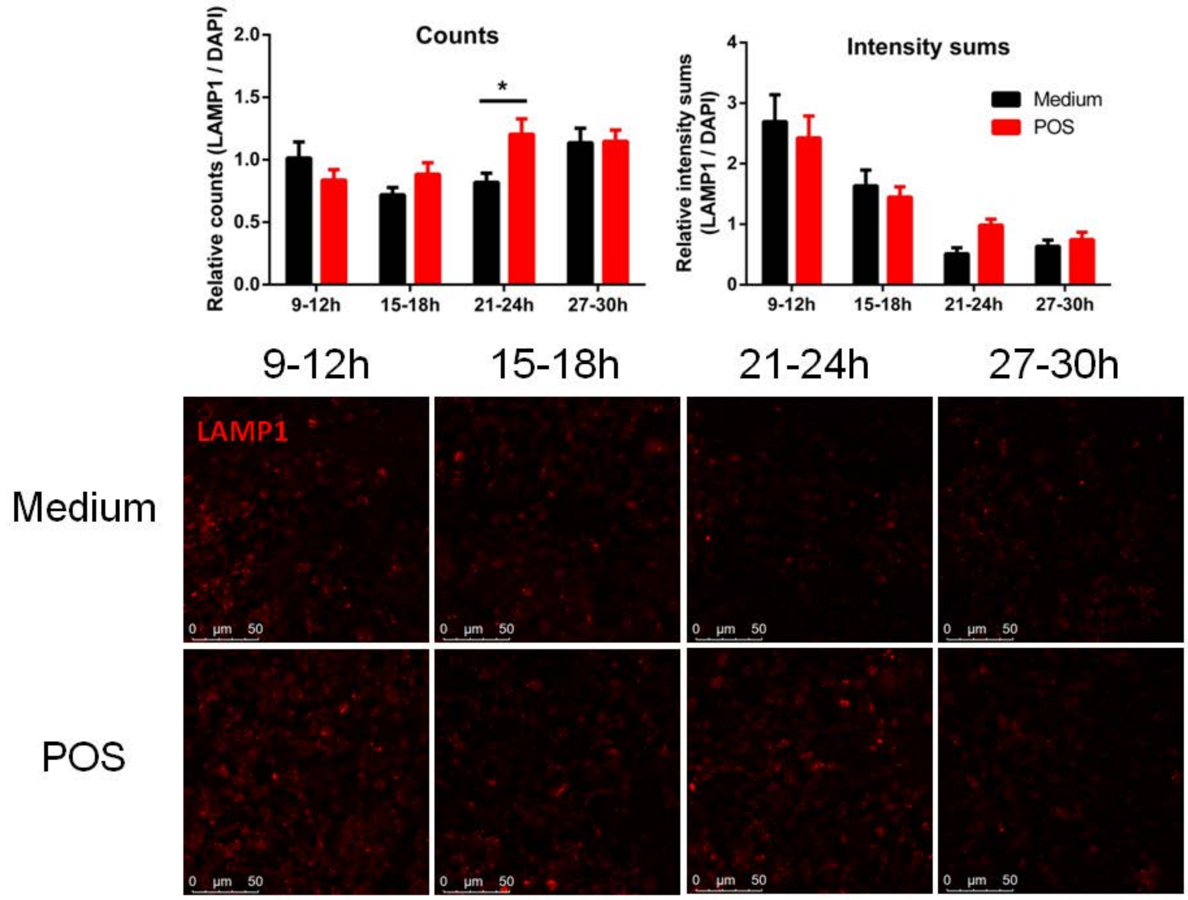

Supplementary figure S5. Circadian regulation of POS processing. ARPE-19 monolayers were serum-shocked (t=0-2h), challenged with bovine POS or medium for 3h at defined time-points and immediately fixed. Lysosomes were visualized with the lysosomal marker LAMP1. (Top) Quantification of z-stacks revealed that time affects the number of LAMP1 positive particles (counts, 2-way ANOVA, $F(3,278) = 2.79$; $p<0.05$) and sums of LAMP1 fluorescence intensities (Intensity sums, 2-way ANOVA, $F(3,287) = 25.82$; $p<0.0001$). Holm-Sidak's post-hoc test revealed that POS incubation induced a higher number of LAMP1 positive particles at t = 21-24h ($p<0.05$). (Bottom) Representative confocal images show that a POS incubation at t = 21-24h induces a stronger LAMP1 signal vs. medium challenged controls. Values are shown as means ± SEM (n = 10 culture inserts / treatment / time-point; total number of z-stacks = 32-42 / treatment / time-point). *$p<0.05$

Uncropped digitally generated WES™ images:

File: 2018-06-11_nm_cn_b-actin

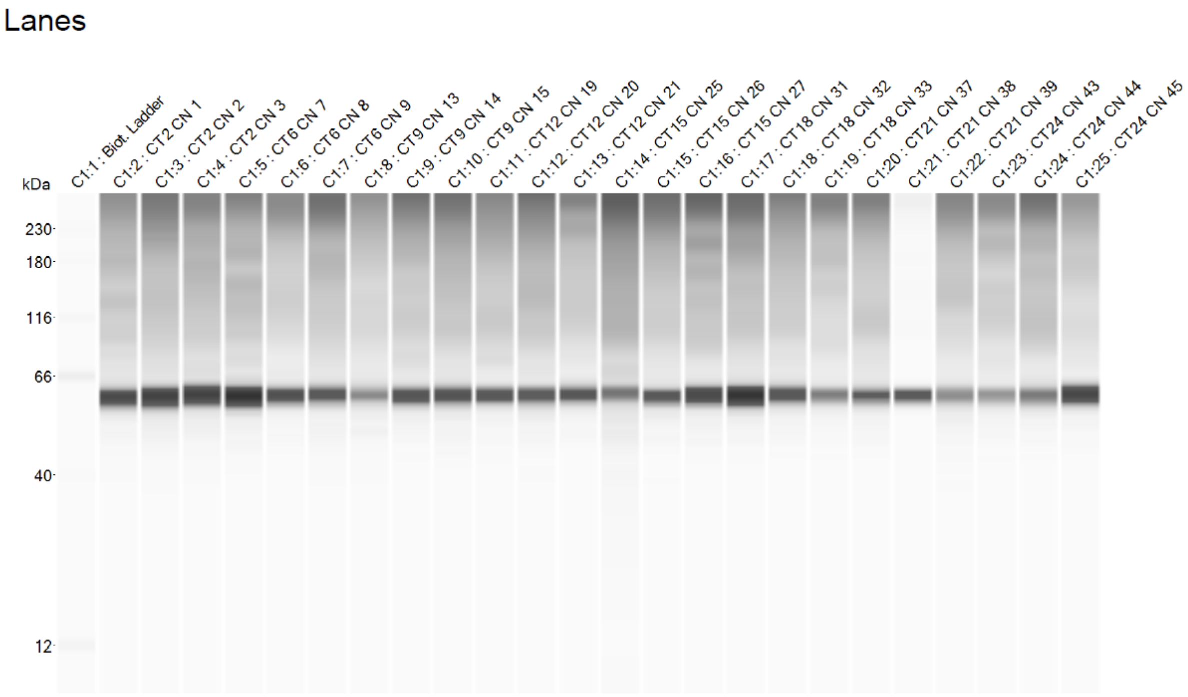

Supplementary figure S6. β-acting protein levels of non-serum-shocked ARPE-19 monolayers.

File: 2018-04-11_project2_exp3_b-actin

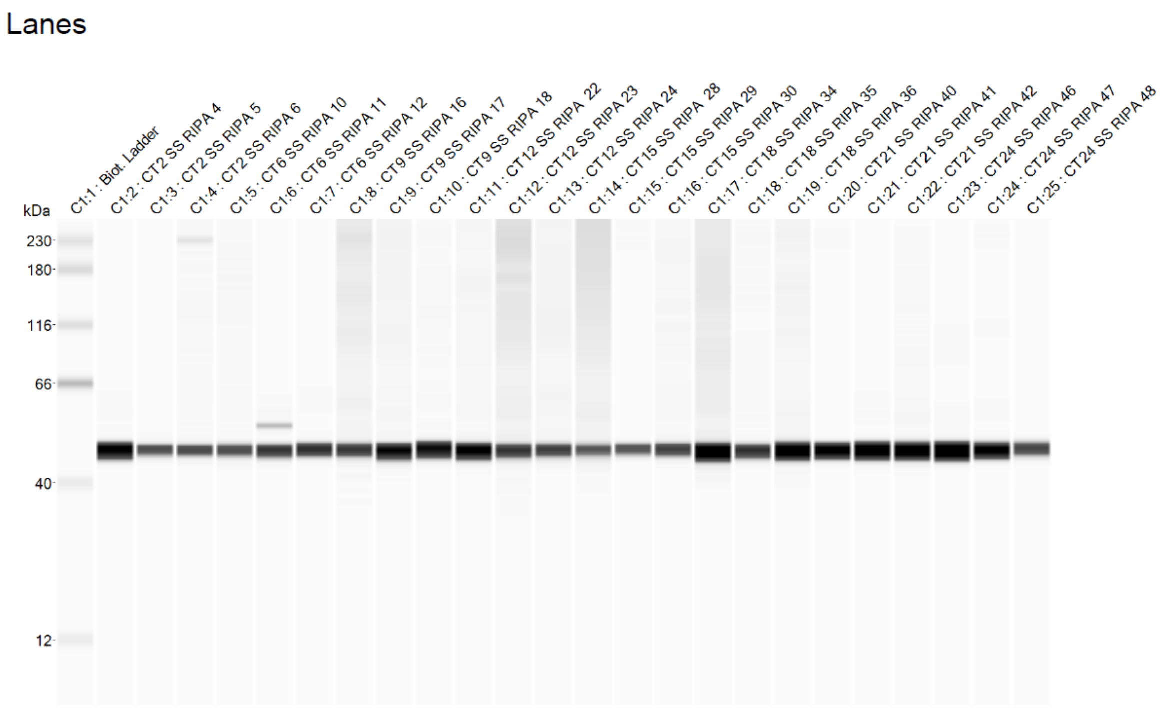

Supplementary figure S7. β-acting protein levels of serum-shocked ARPE-19 monolayers.

File: 2018-06-06_nm_project2_exp3_fak_cn

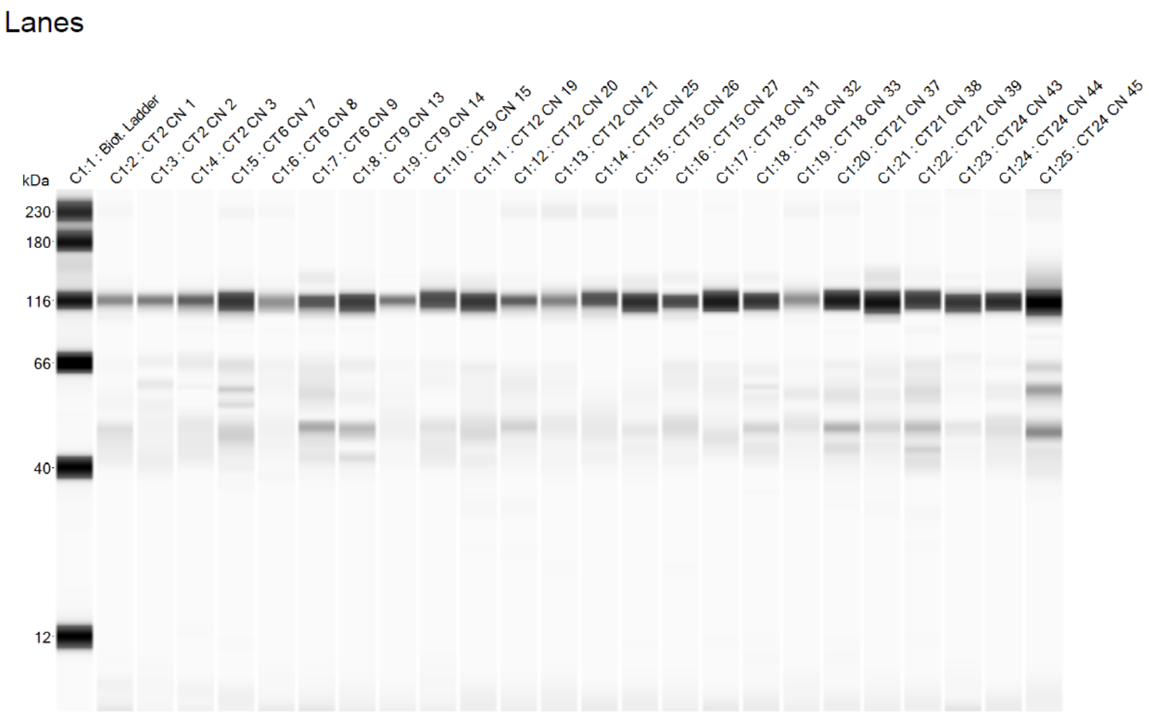

Supplementary figure S8. FAK protein levels of non-serum-shocked ARPE-19 monolayers.

File: 2018-04-05_nm fak serum shocked arpe-19

Lanes

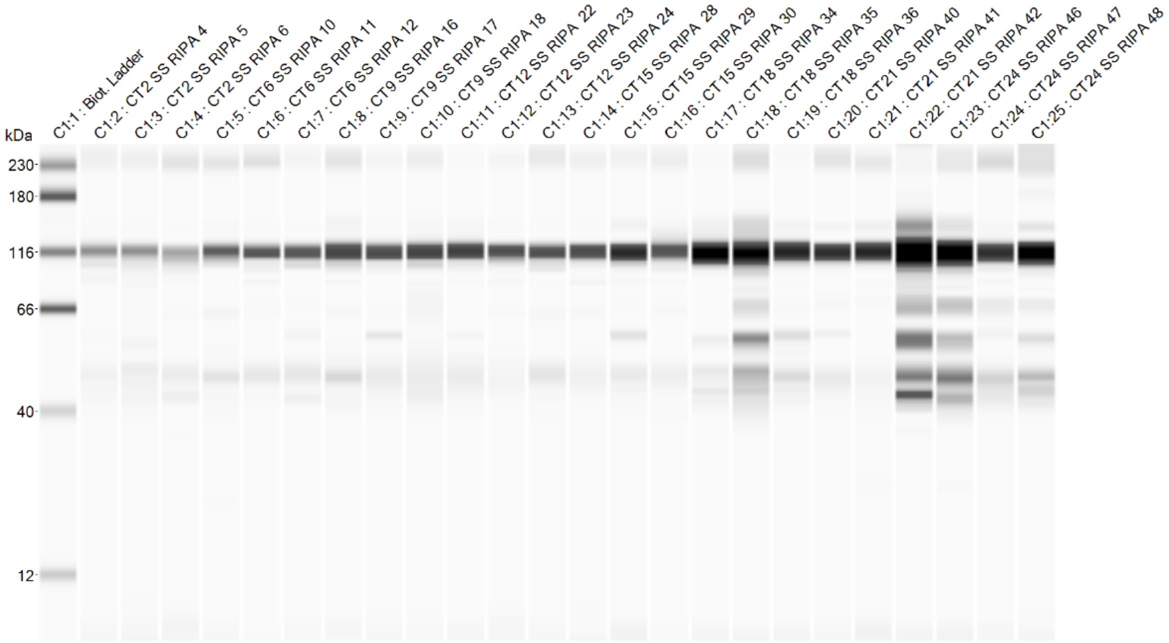

Supplementary figure S9. FAK protein levels of serum-shocked ARPE-19 monolayers.

File: 2018-06-05_nm_exp3_cn_itgb5

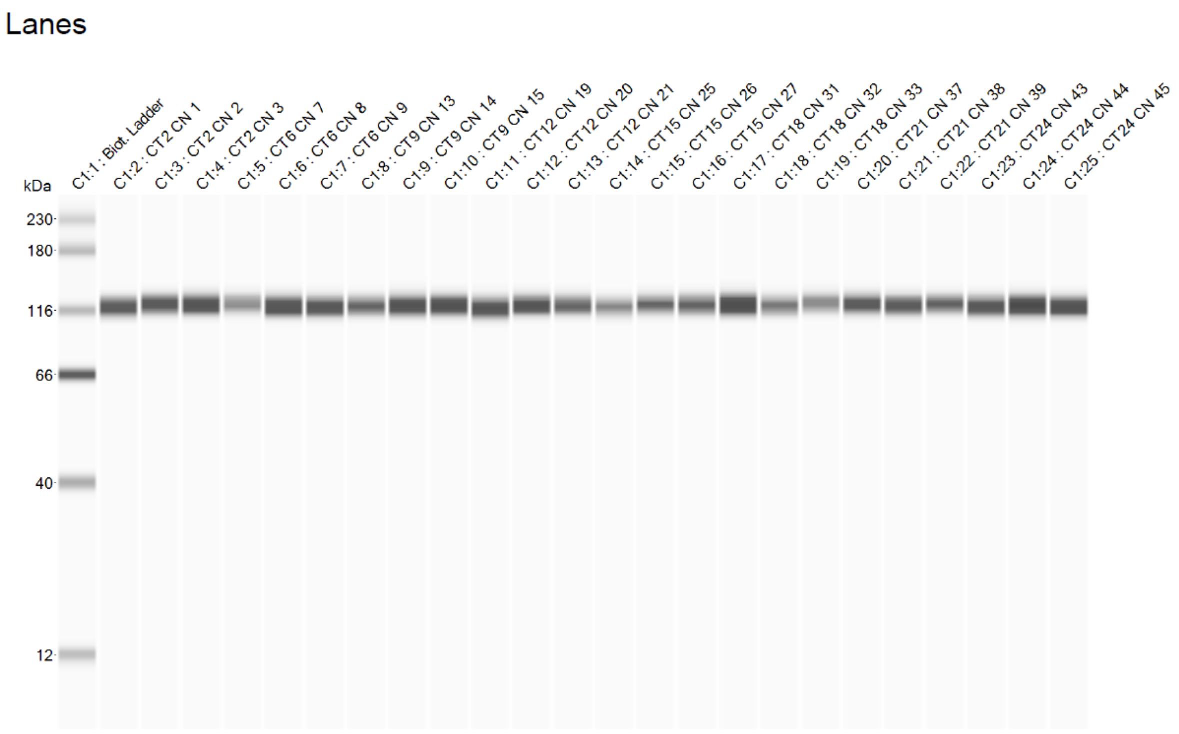

Supplementary figure S10. ITGB5 protein levels of non-serum-shocked ARPE-19 monolayers.

File: 2018-04-17_project2_exp3_itgb5

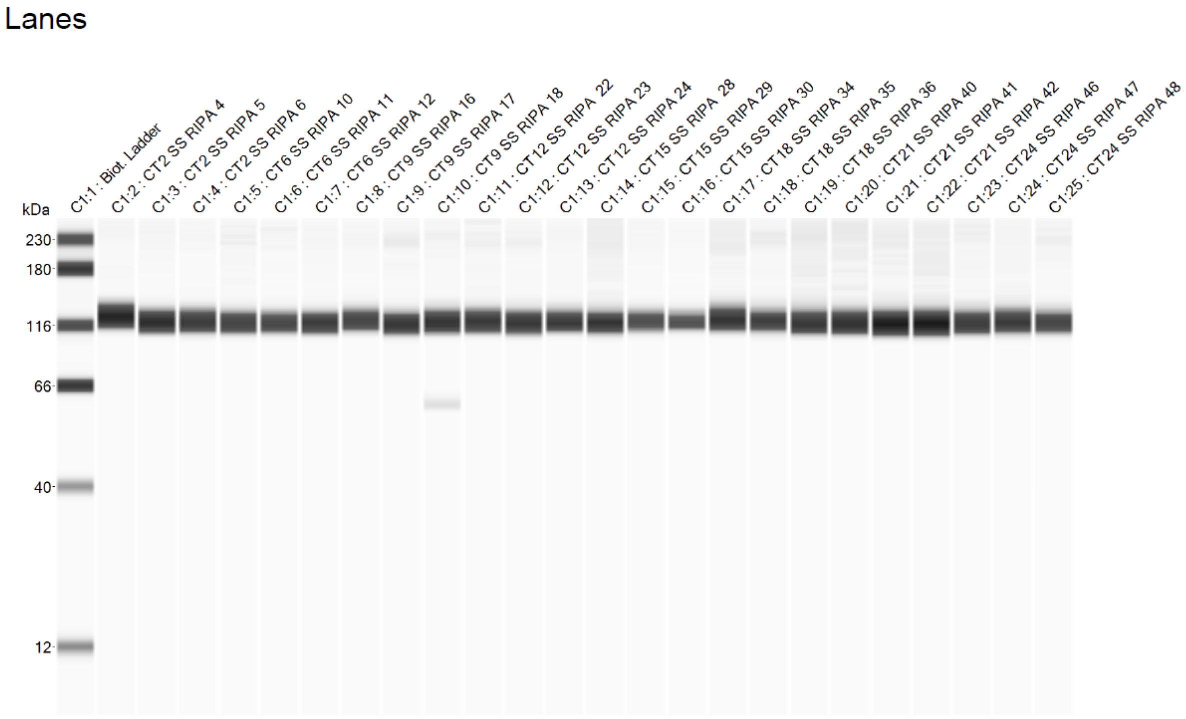

Supplementary figure S11. ITGB5 protein levels of serum-shocked ARPE-19 monolayers.

File: 2018-04-09_nm_pros_testing_gapdh_b-actin

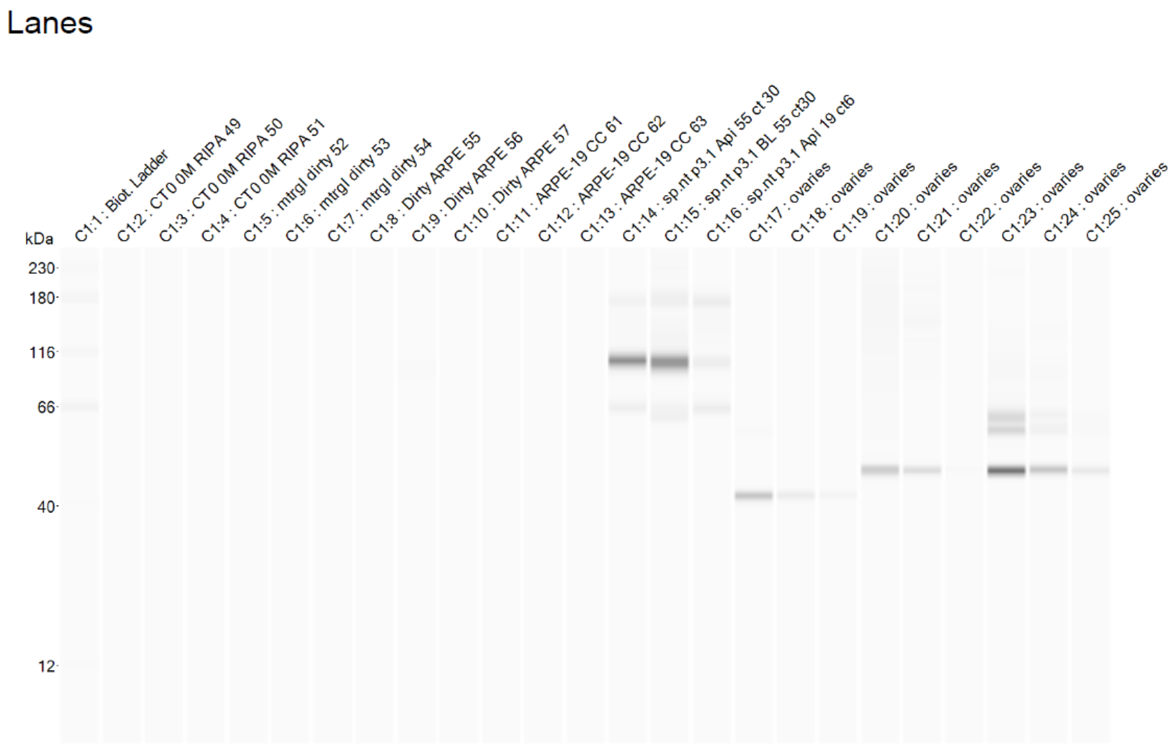

Supplementary figure S12. Protein S protein levels in various ARPE-19 culturing conditions. The remaining WES plate wells were used for testing antibodies.

Images of gel electrophoresis of PCR products used for quantification in Figure 1. Samples are labelled on the gel (n = 3 per time point):

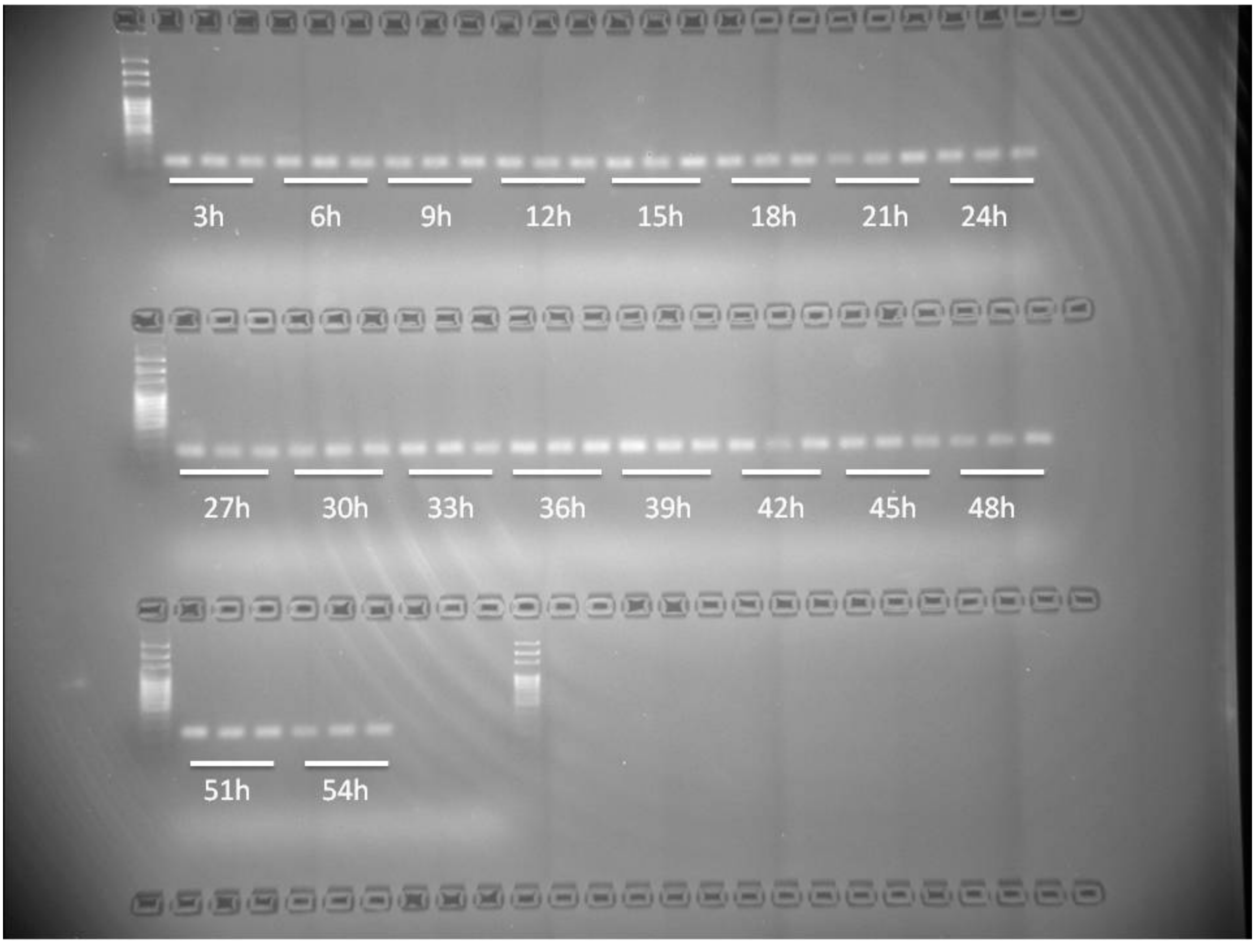

Supplementary figure S13. Gel electrophoresis of *EF1α* PCR products of dispersed ARPE-19 cell cultures.

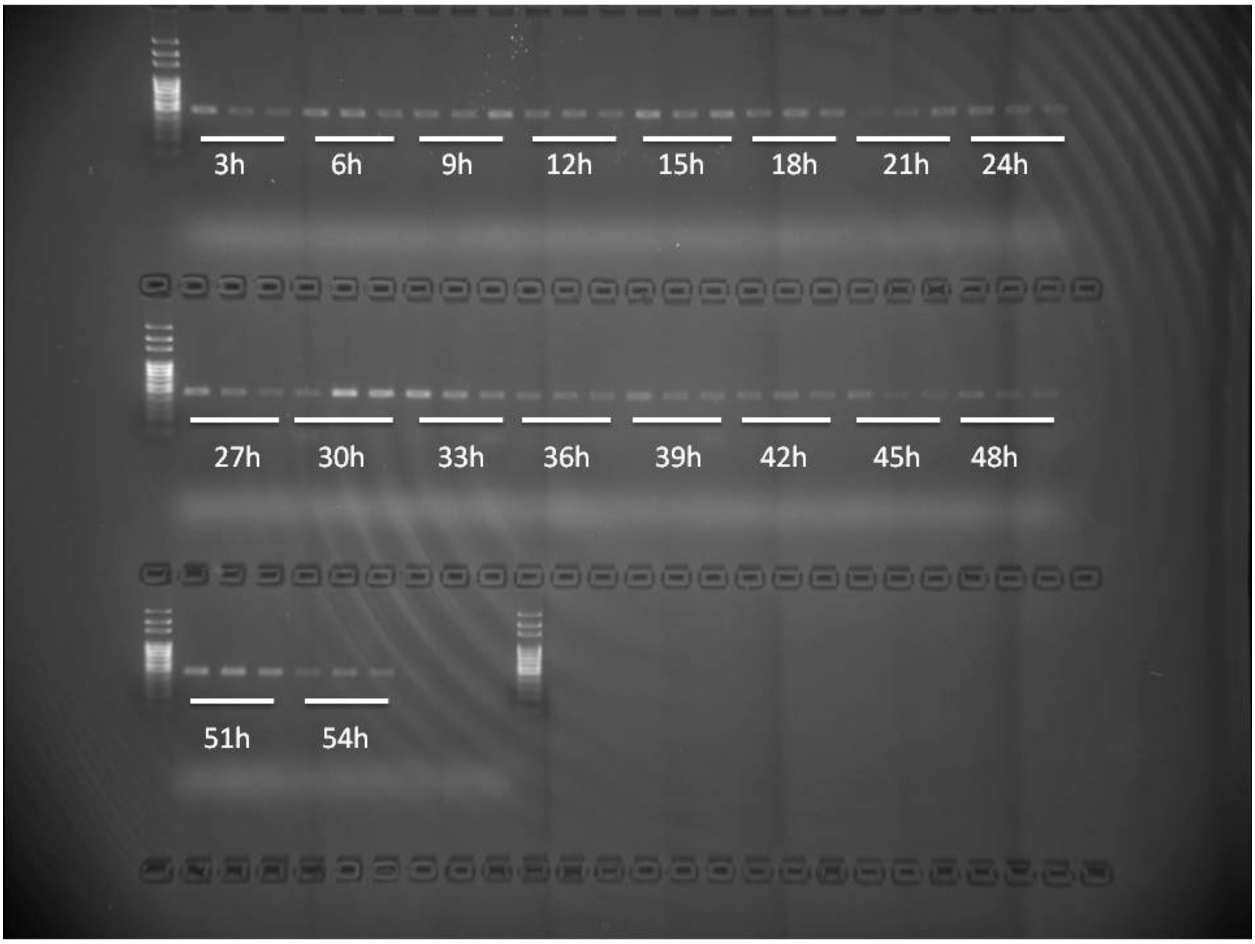

Supplementary figure S14. Gel electrophoresis of *ARNTL* PCR products of dispersed ARPE-19 cell cultures.

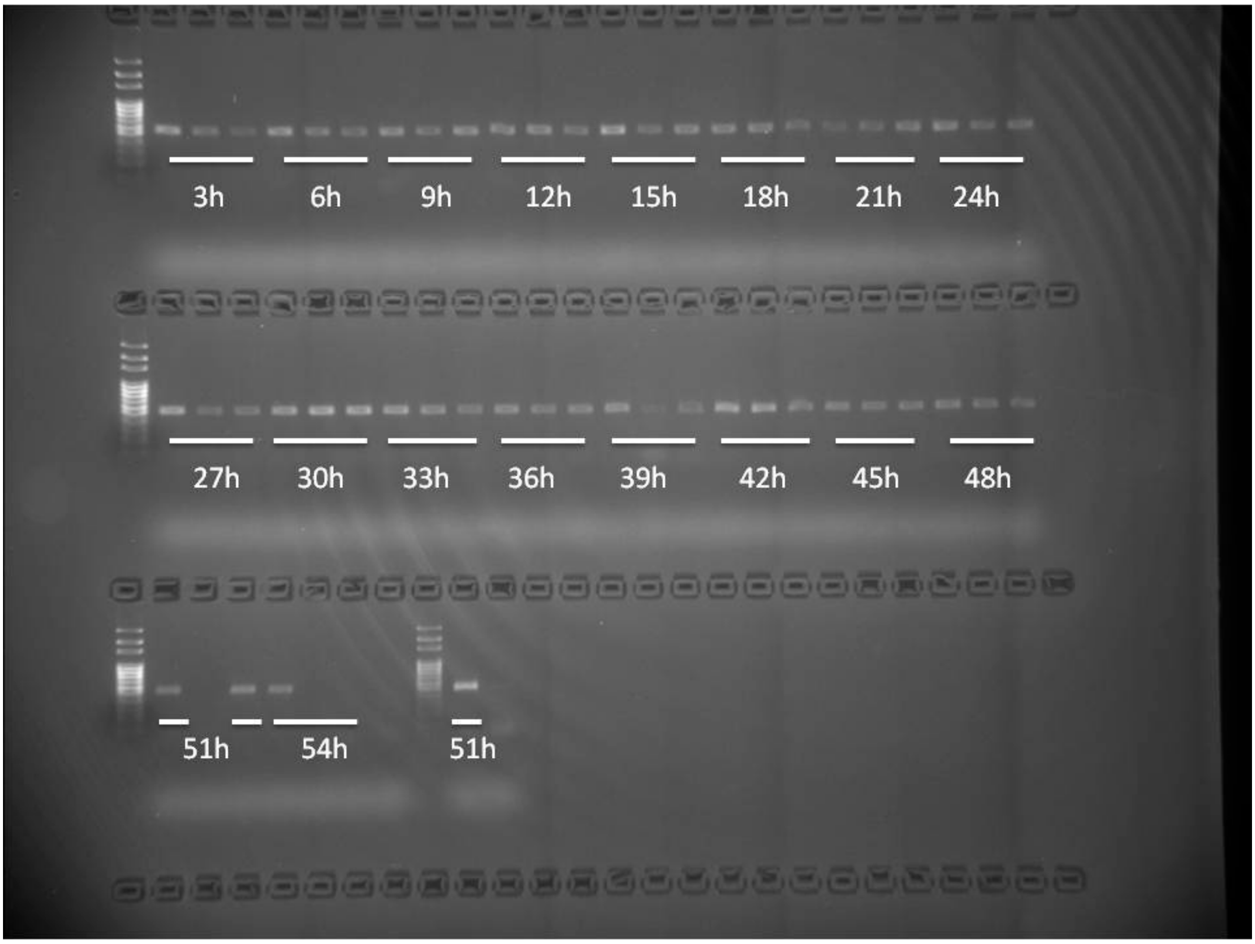

Supplementary figure S15. Gel electrophoresis of *CLOCK* PCR products of dispersed ARPE-19 cell cultures. The missing sample in the third row is pipetted after the ladder due to an error.

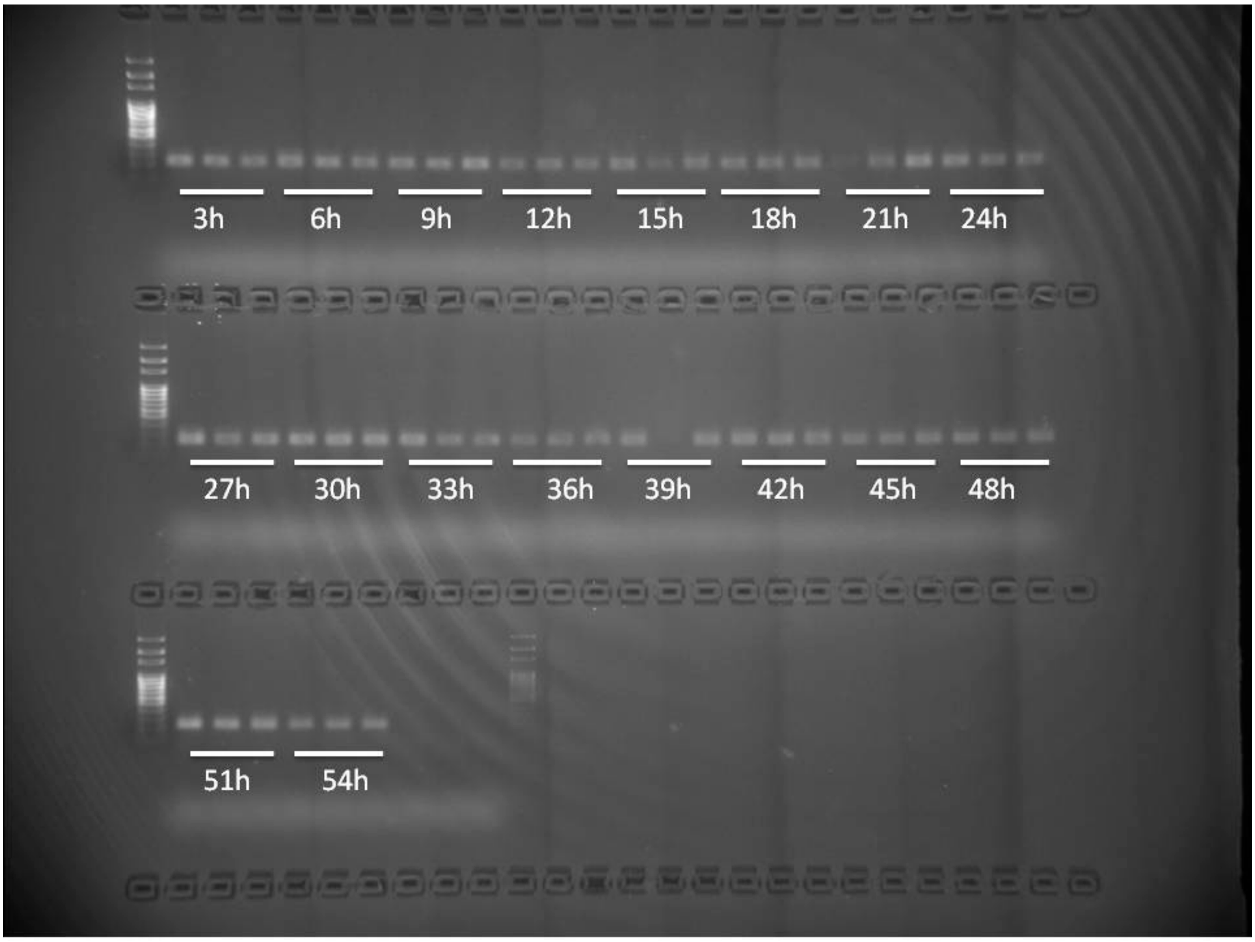

Supplementary figure S16. Gel electrophoresis of *CRY1* PCR products of dispersed ARPE-19 cell cultures.

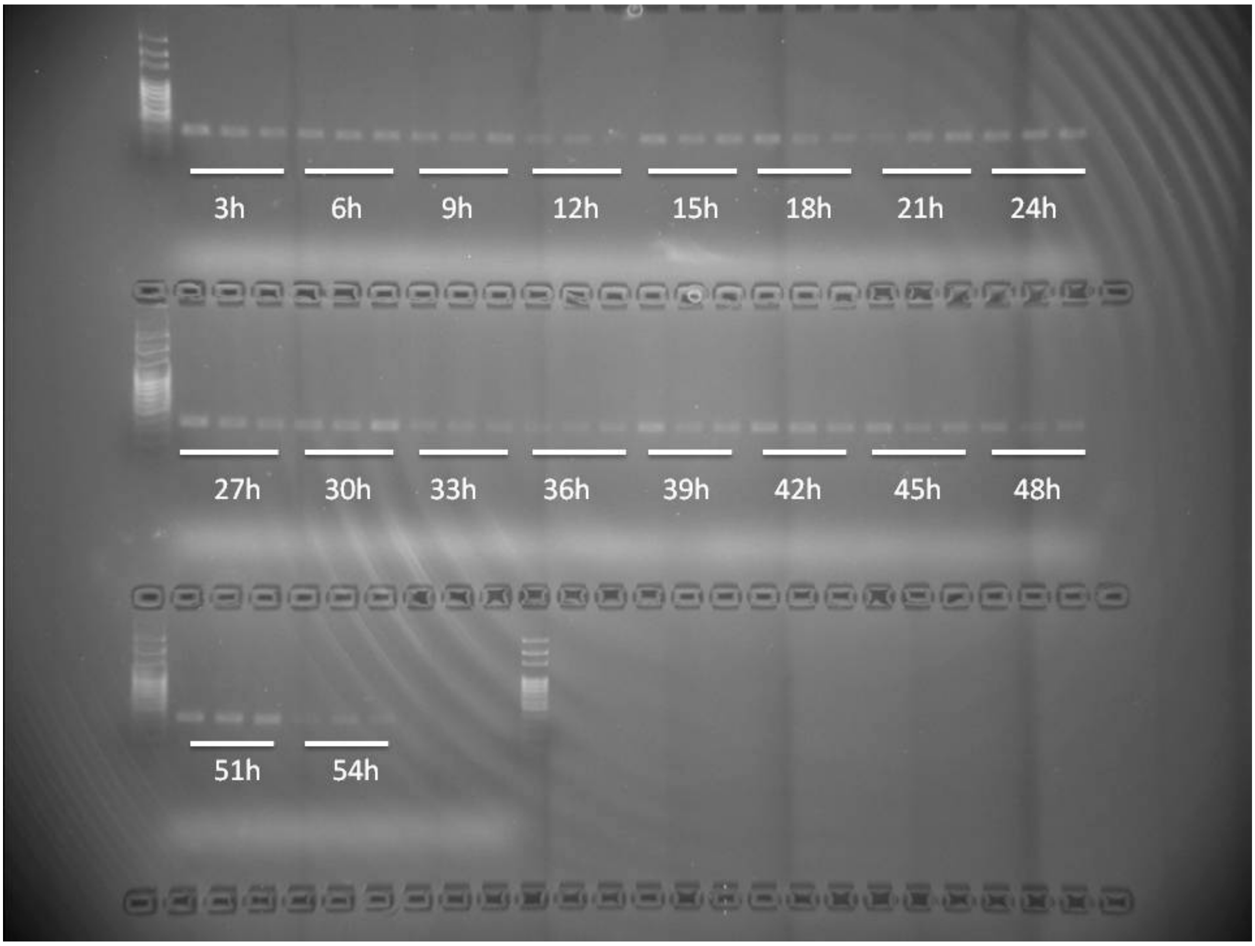

Supplementary figure S17. Gel electrophoresis of *CRY2* PCR products of dispersed ARPE-19 cell cultures.

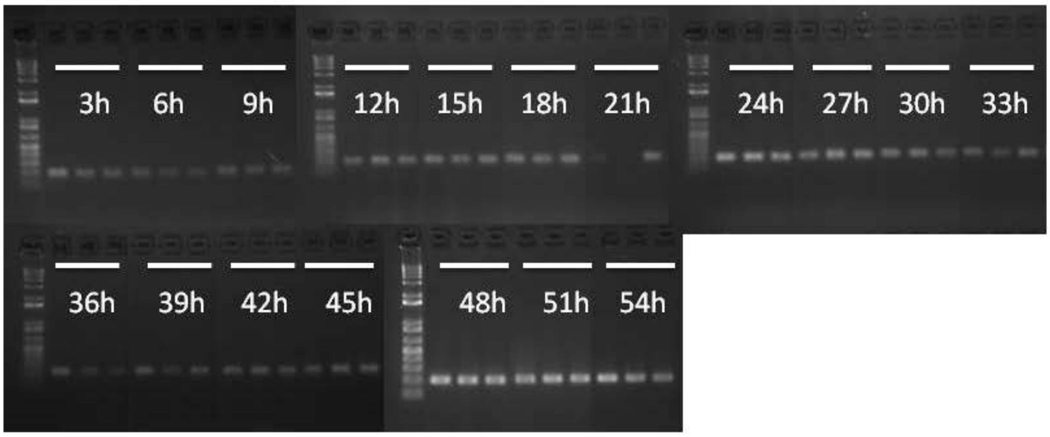

Supplementary figure S18. Gel electrophoresis of *PER1* PCR products of dispersed ARPE-19 cell cultures. The gel contained samples from other experiments and was cropped. The resulting images were merged and contain only samples used for quantification.

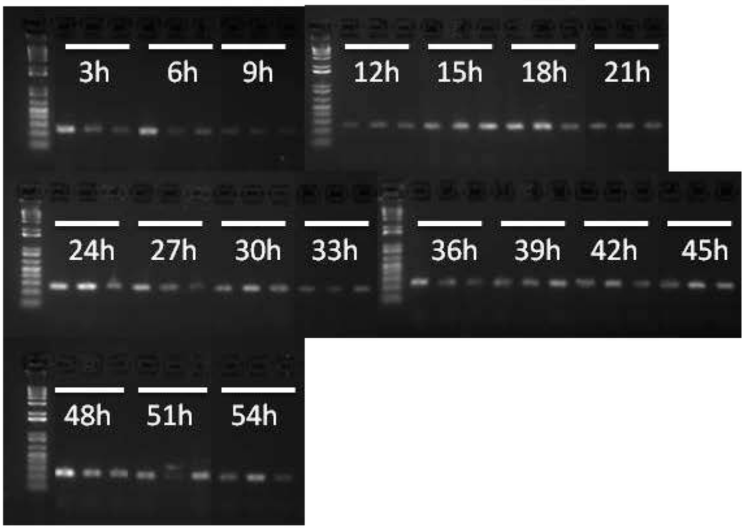

Supplementary figure S19. Gel electrophoresis of *PER2* PCR products of dispersed ARPE-19 cell cultures. The gel contained samples from other experiments and was cropped. The resulting images were merged and contain only samples used for quantification.

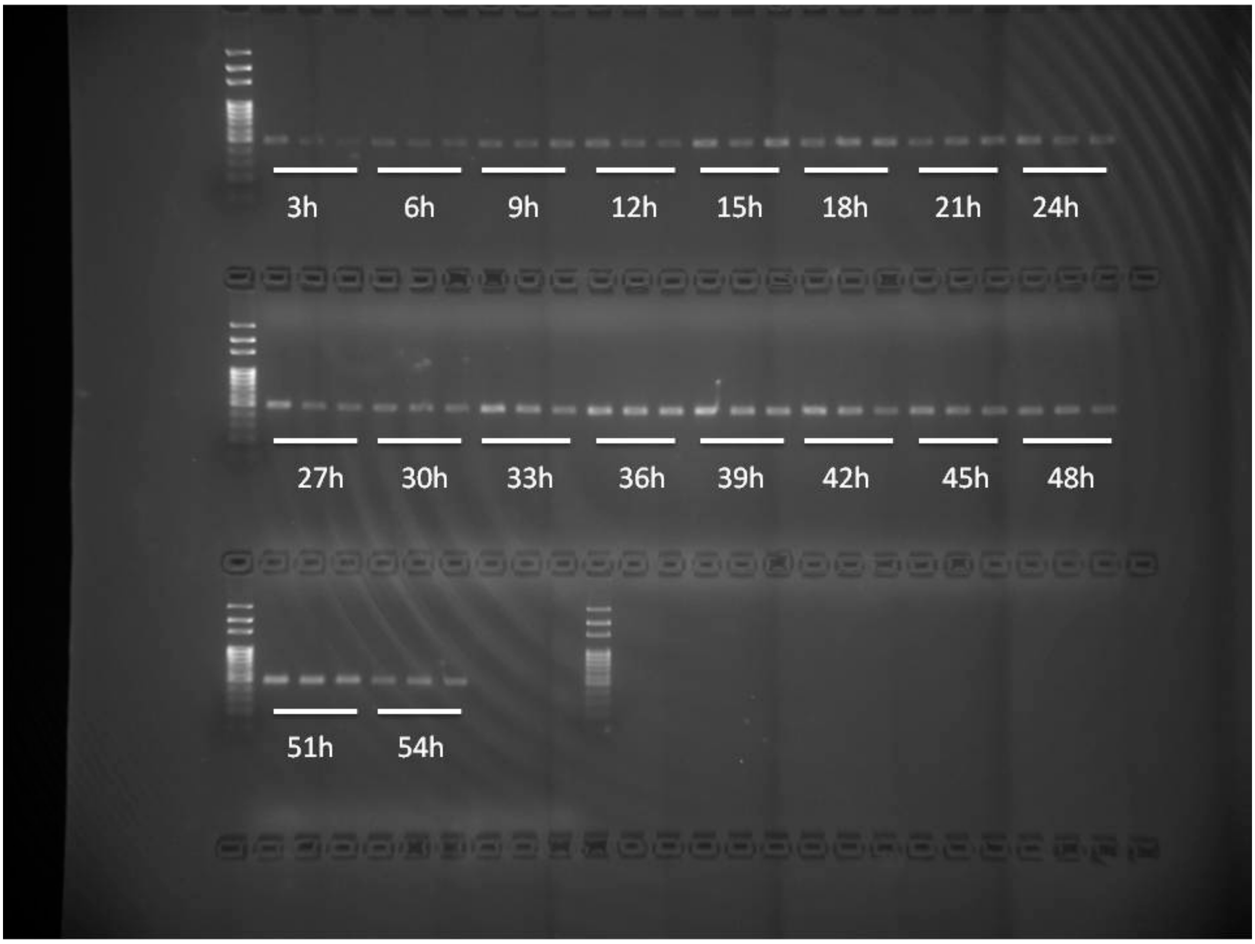

Supplementary figure S20. Gel electrophoresis of *REV-ERB*α PCR products of dispersed ARPE-19 cell cultures.

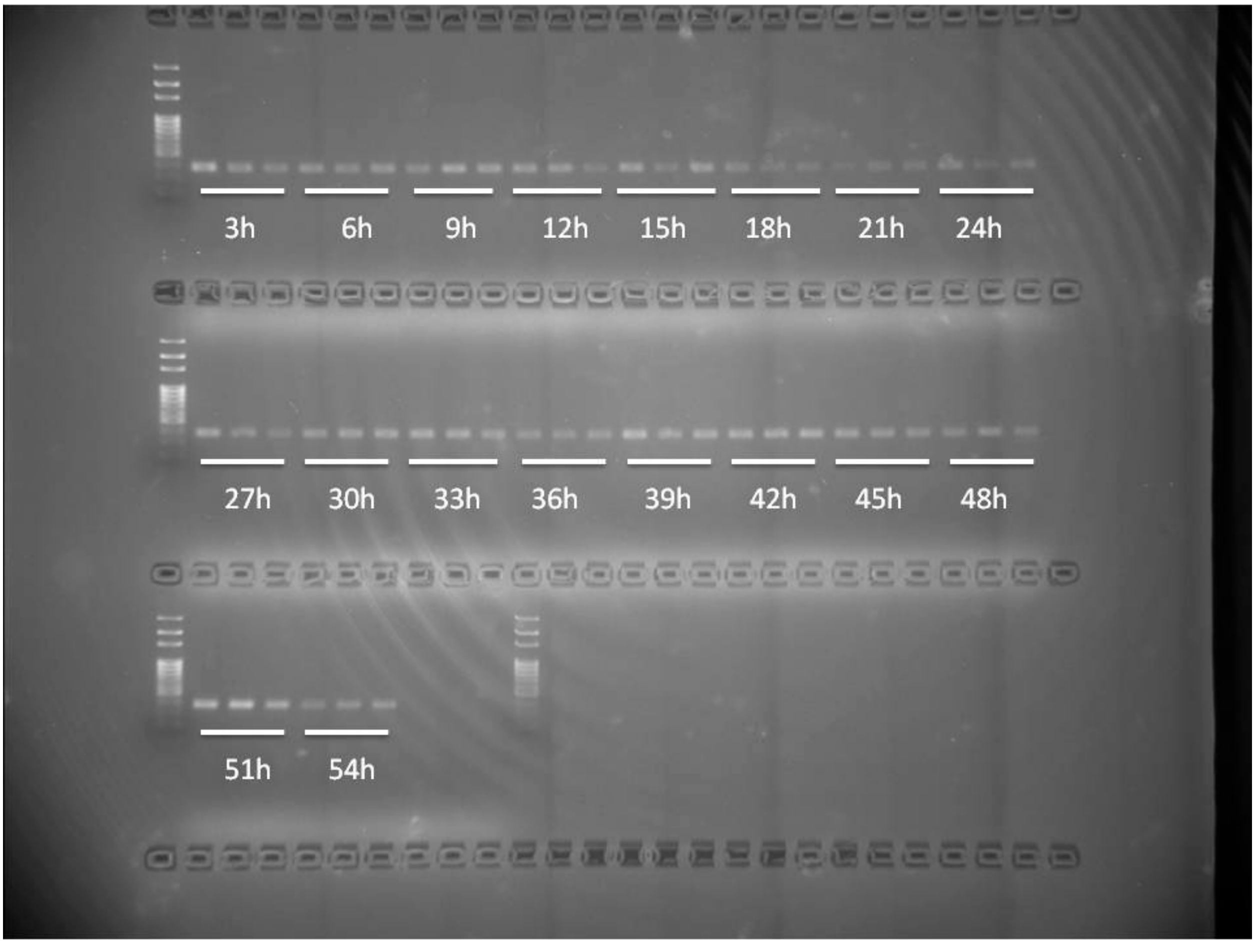

Supplementary figure S21. Gel electrophoresis of *GAS6* PCR products of dispersed ARPE-19 cell cultures.

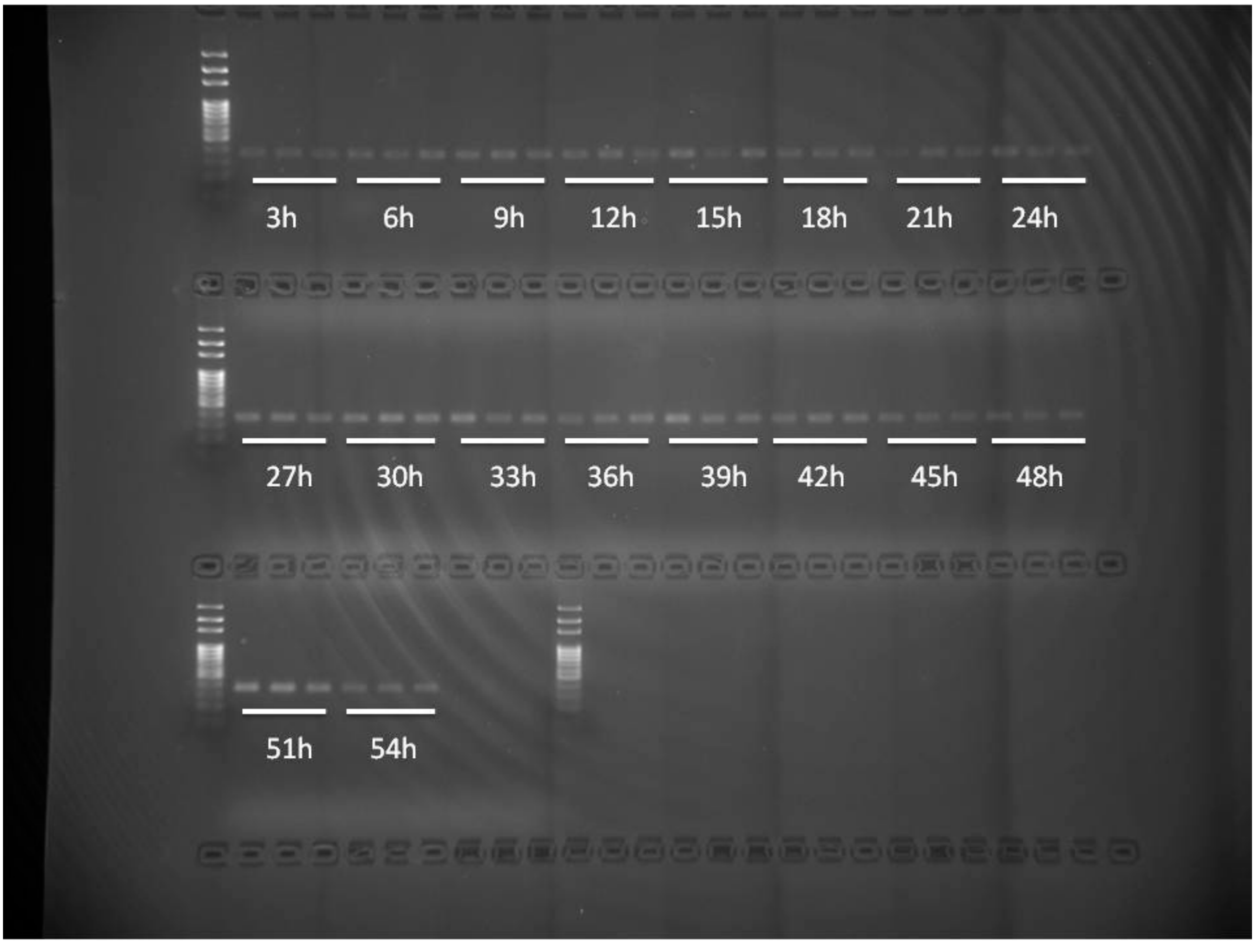

Supplementary figure S22. Gel electrophoresis of *ITGB5* PCR products of dispersed ARPE-19 cell cultures.

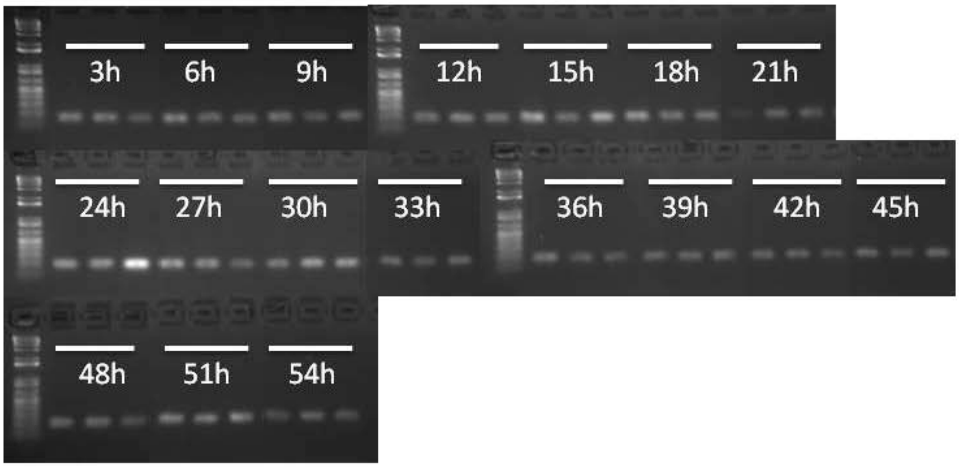

Supplementary figure S23. Gel electrophoresis of *LAMP1* PCR products of dispersed ARPE-19 cell cultures. The gel contained samples from other experiments and was cropped. The resulting images were merged and contain only samples used for quantification.

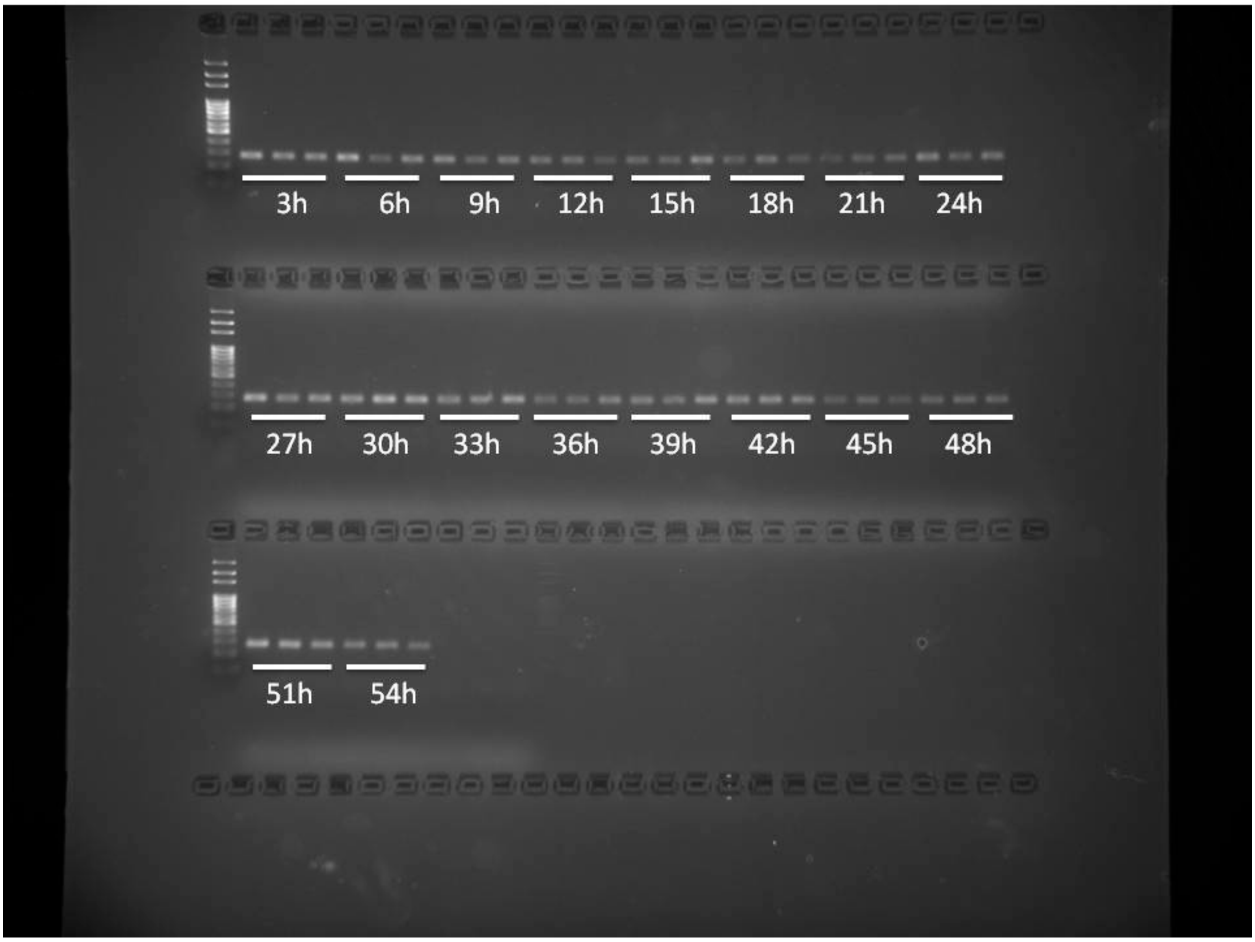

Supplementary figure S24. Gel electrophoresis of *MFGE8* PCR products of dispersed ARPE-19 cell cultures.

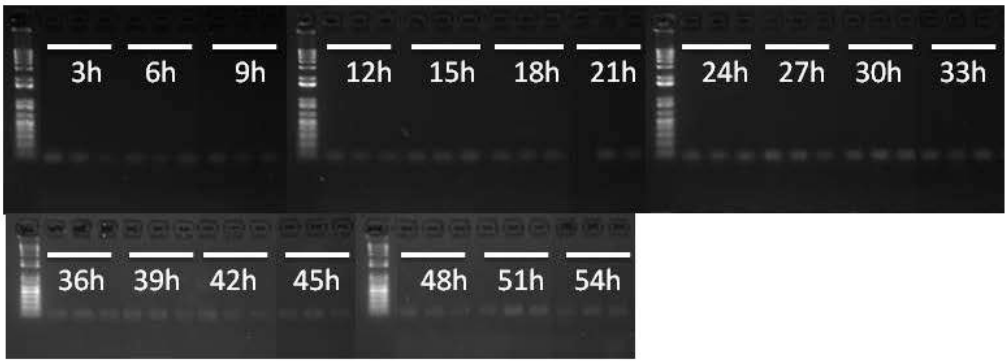

Supplementary figure S25. Gel electrophoresis of *PROS1* PCR products of dispersed ARPE-19 cell cultures. The gel contained samples from other experiments and was cropped. The resulting images were merged and contain only samples used for quantification.

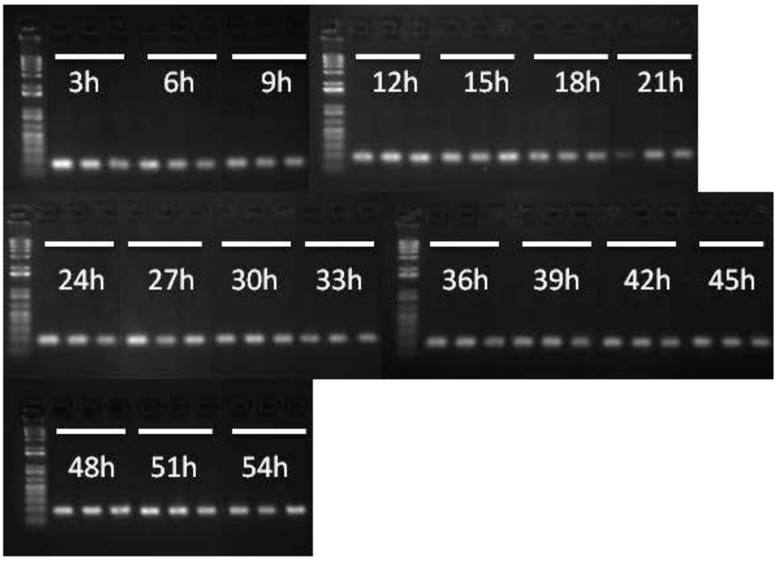

Supplementary figure S26. Gel electrophoresis of *PTK2* PCR products of dispersed ARPE-19 cell cultures. The gel contained samples from other experiments and was cropped. The resulting images were merged and contain only samples used for quantification.

Images of gel electrophoresis of PCR products used for quantification in Figure 2. Samples are labelled on the gels (n = 3 per time point). Gels with samples from other experiments were cropped out. The resulting images were merged.

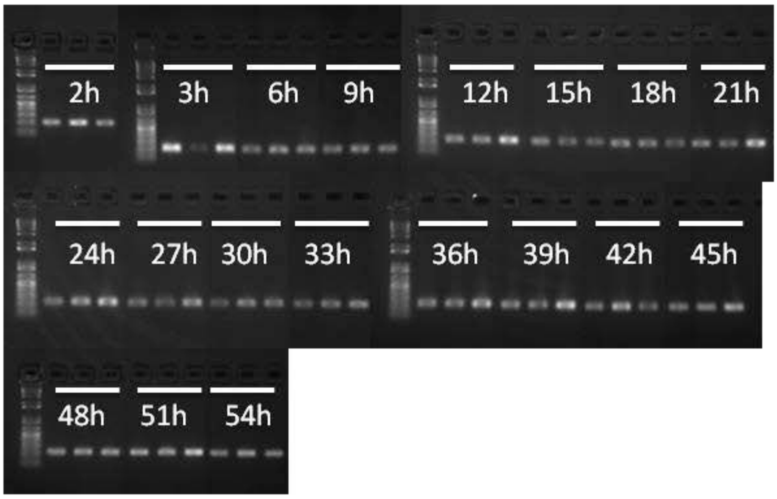

Supplementary figure S27. Gel electrophoresis of *EF1α* PCR products of ARPE-19 monolayer cell cultures.

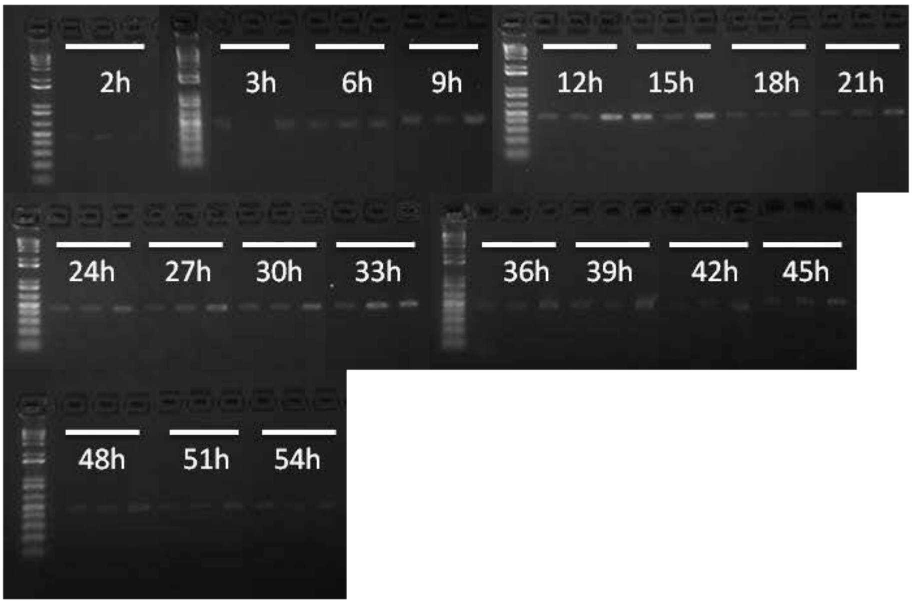

Supplementary figure S28. Gel electrophoresis of *ARNTL* PCR products of ARPE-19 monolayer cell cultures.

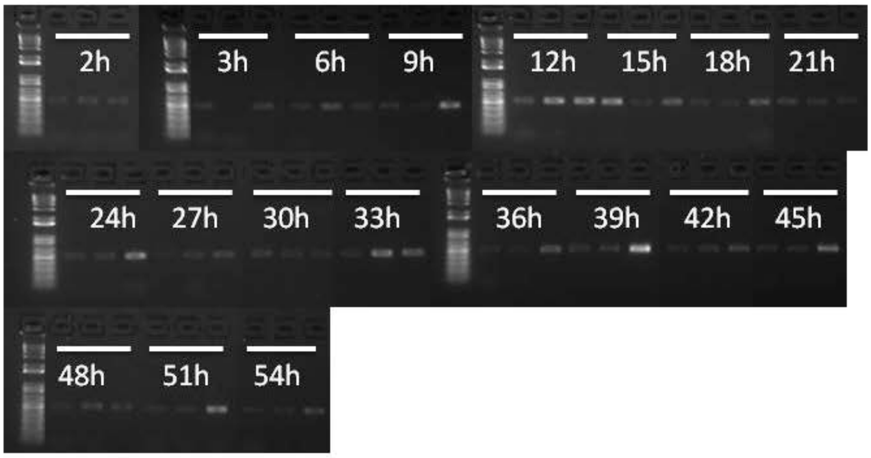

Supplementary figure S29. Gel electrophoresis of *CLOCK* PCR products of ARPE-19 monolayer cell cultures.

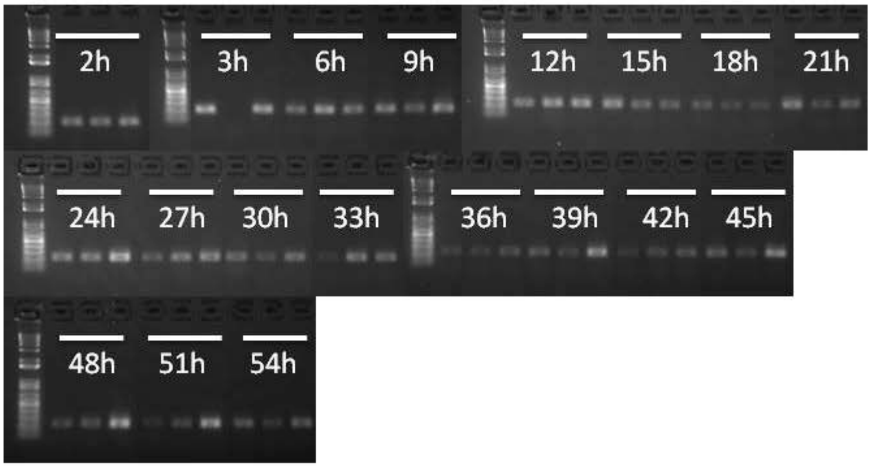

Supplementary figure S30. Gel electrophoresis of *CRY1* PCR products of ARPE-19 monolayer cell cultures.

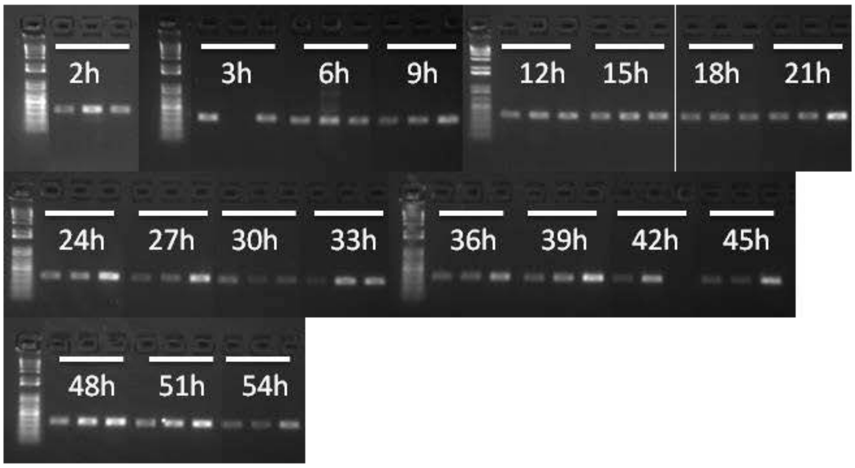

Supplementary figure S31. Gel electrophoresis of *CRY2* PCR products of ARPE-19 monolayer cell cultures

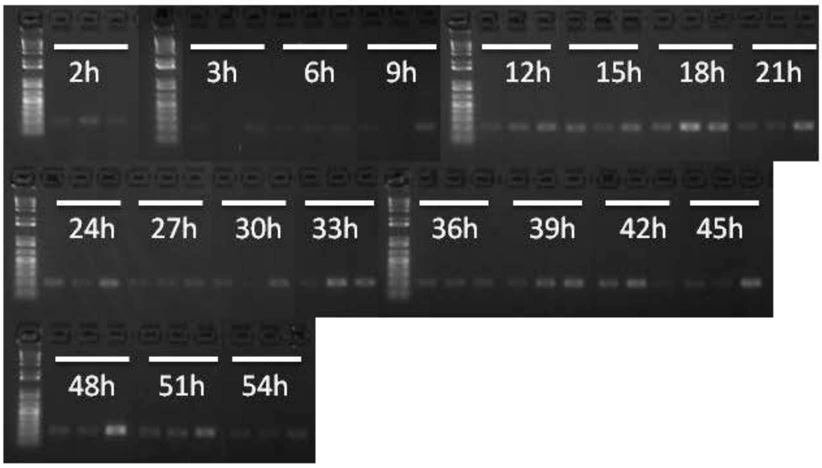

.

Supplementary figure S32. Gel electrophoresis of *PER1* PCR products of ARPE-19 monolayer cell cultures.

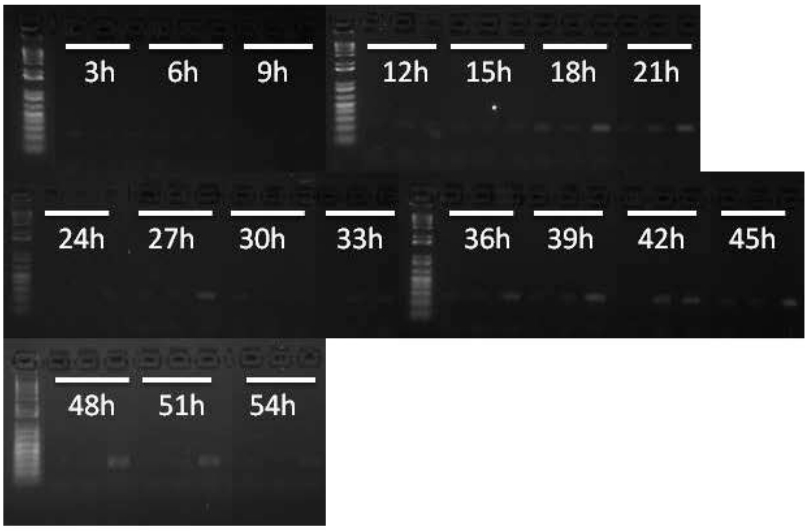

Supplementary figure S33. Gel electrophoresis of *PER2* PCR products of ARPE-19 monolayer cell cultures.

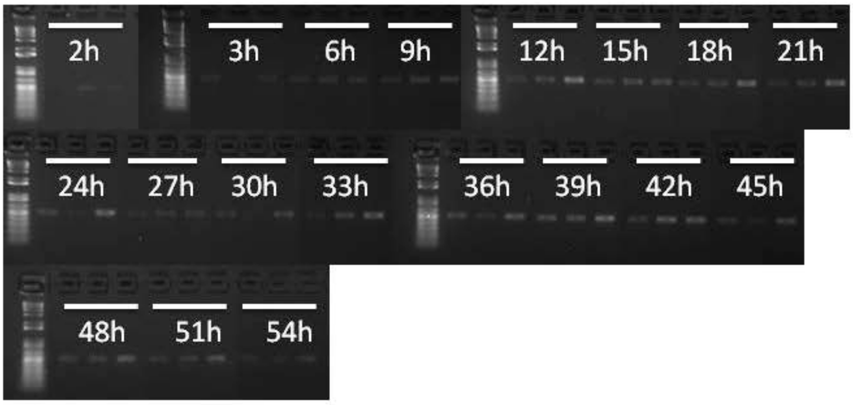

Supplementary figure S34. Gel electrophoresis of *REV-ERB*α PCR products of ARPE-19 monolayer cell cultures.

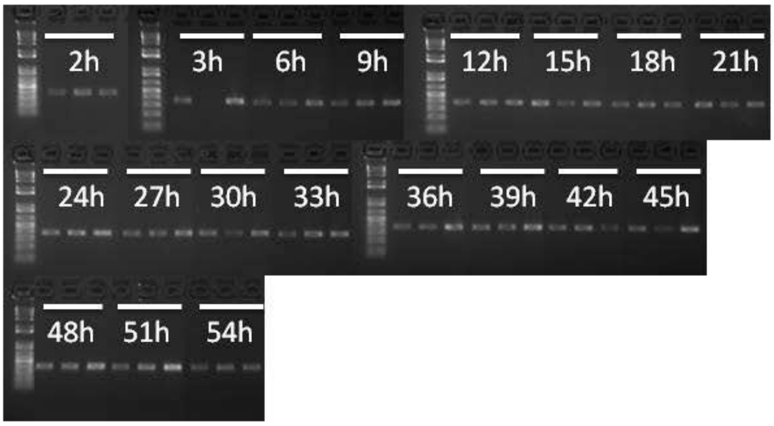

Supplementary figure S35. Gel electrophoresis of *GAS6* PCR products of ARPE-19 monolayer cell cultures.

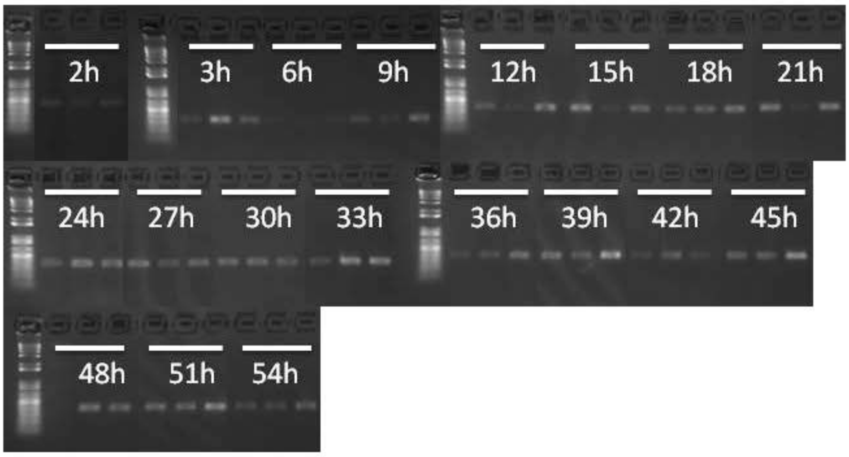

Supplementary figure S36. Gel electrophoresis of *ITGB5* PCR products of ARPE-19 monolayer cell cultures.

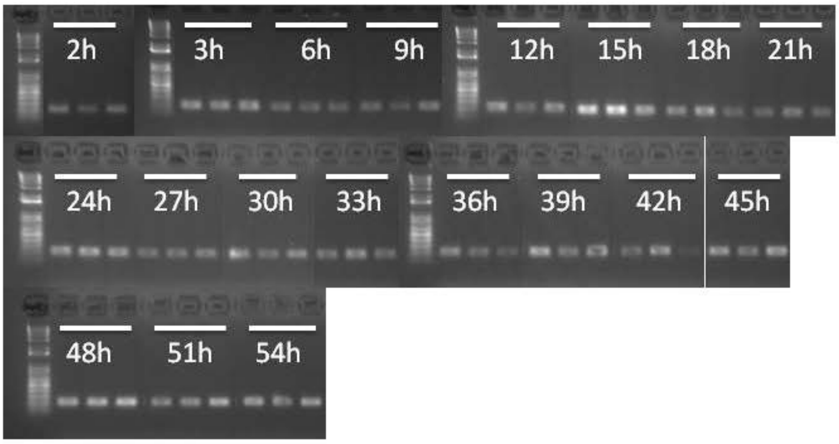

Supplementary figure S37. Gel electrophoresis of *LAMP1* PCR products of ARPE-19 monolayer cell cultures.

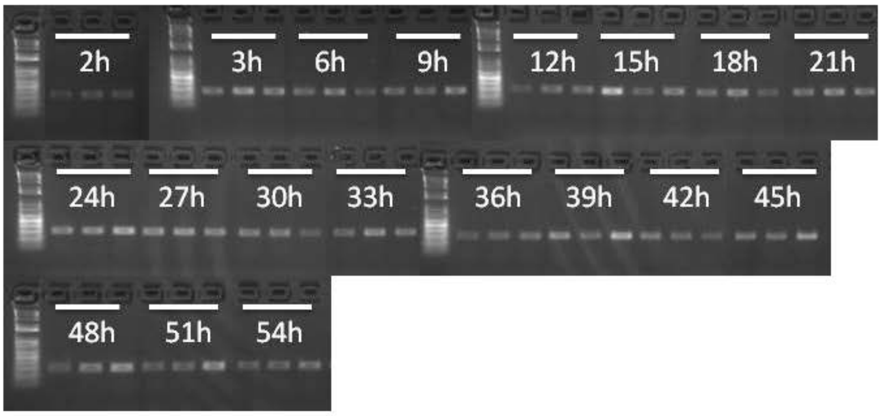

Supplementary figure S38. Gel electrophoresis of *MFGE8* PCR products of ARPE-19 monolayer cell cultures.

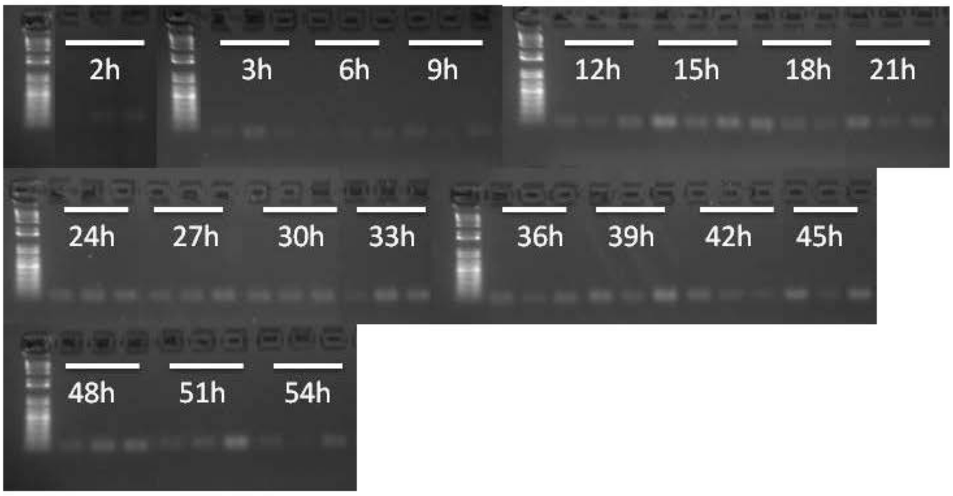

Supplementary figure S39. Gel electrophoresis of *PROS1* PCR products of ARPE-19 monolayer cell cultures.

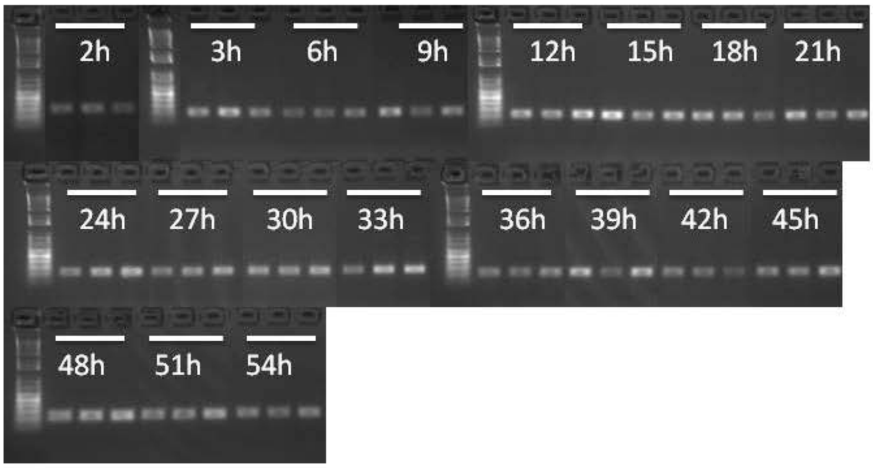

Supplementary figure S40. Gel electrophoresis of *PTK2* PCR products of ARPE-19 monolayer cell cultures.
